# Dynamic Covalent Boronate Chemistry for *In Situ* Formation, Interfacial Stabilization, and Cytomimetic Optimization of Coacervates

**DOI:** 10.1021/jacs.5c17688

**Published:** 2026-02-27

**Authors:** Bruno Delgado Gonzalez, Lucas Garcia-Abuin, Celia Jimenez-Lopez, Eduardo Fernandez-Megia

**Affiliations:** Centro Singular de Investigación en Química Biolóxica e Materiais Moleculares (CIQUS), Departamento de Química Orgánica, Universidade de Santiago de Compostela, Jenaro de la Fuente s/n, 15782 Santiago de Compostela, Spain

## Abstract

Bioinspired synthetic cells are rapidly transforming the way we interrogate the principles of cellular life and the development of bioengineering and medical applications. However, despite significant progress in modeling cell-like behavior, material engineering remains a time-consuming and often behind-the-scenes endeavor when optimizing cytomimetic functions. Here, we describe how dynamic covalent chemistry can be used to bypass this bottleneck using membranized coacervate microdroplets (MCM) as synthetic cell models. Specifically, the potential of dynamic covalent boronate chemistry for the *in situ* formation, interfacial stabilization, and adaptive cytomimetic optimization of MCM is presented. Simultaneous addition of cationic and anionic catechols to a polymeric boronic acid (BA) generates dynamic zwitterionic polyboronates that spontaneously phase separate into microdroplets, which can then be interfacially stabilized as MCM with a BA-functionalized block copolymer. The cytomimetic properties, membranization, internal dynamics, and enzymatic activity within the MCM can be modulated *in situ* using dynamic covalent libraries to fine-tune material properties (either by adjusting the charge ratio between oppositely charged catechols, varying the catechol-to-BA ratio, or introducing auxiliary catechol dopants) without the need to synthesize, isolate, purify, and characterize new polymeric materials. Application of this technology to other catechols, multivalent BA, and synthetic cell architectures holds promise for optimizing diverse biomimetic functions and providing programmable synthetic cells with emerging properties.

## Graphical abstract

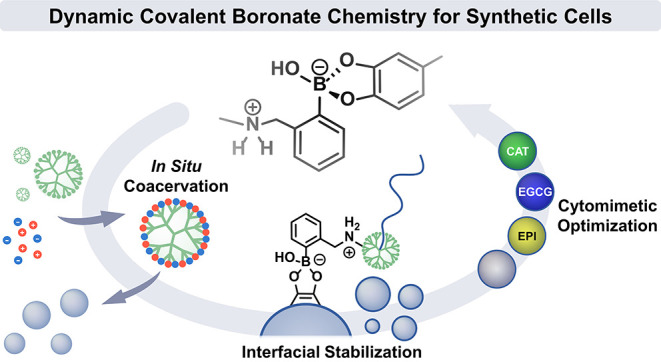

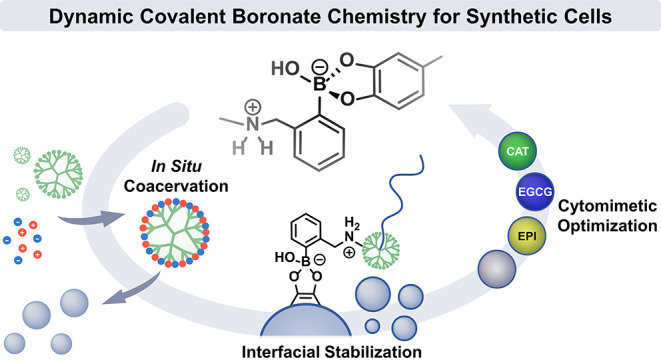

## Introduction

Efforts to understand the compartmentalization and evolution of natural cells have fueled the rise of bottom-up synthetic biology,^1^ which harnesses bioinspired synthetic cell models^2–4^ to shed light on primitive forms of life and enable innovative applications in bioengineering and medicine.^5–8^ As the membrane is a hallmark of natural cells,^9^ artificial vesicles have attracted the most attention as synthetic cell models exhibiting typical cellular functions. However, their inability to reproduce the molecularly crowded interior of eukaryotic cells^10^ has prompted the development of alternative models. Among them, complex coacervates are compelling candidates.^11,12^ These microdroplets, formed in water by spontaneous phase separation of oppositely charged polyelectrolytes,^13,14^ contain an internal aqueous phase enriched in low molecular weight compounds and biomolecules sequestered from the surrounding diluted milieu, thereby constituting a truthful mimic of the complex interior of cells^15–18^ and membraneless organelles.^19–21^ Despite the structural diversity of coacervates supporting a wide range of biomimetic functions, their lack of an enclosing membrane remains a major drawback when compared to vesicles, as coacervates tend to readily coalesce. This compromises their long-term stability and potential applications. Interfacial stabilization has recently emerged as a powerful way to circumvent this limitation using auxiliary components assembled on the surface of the microdroplets: fatty acids,^22^ phospholipids,^23^ liposomes,^24^ terpolymers,^25,26^ polysaccharides,^27^ dendrimers,^28^ inorganic nanoparticles,^29^ metal-phenolic networks,^30^ proteins,^31^ membrane fragments,^32^ and even living bacteria.^33^ These hybrid systems, known as membranized coacervate microdroplets (MCM), integrate both vesicle and coacervate cell models into a single construct.^34^

When compared to living cells, which have evolved for millions of years, complex coacervates and MCM are still in their infancy in modeling cell-like behavior and functions, such as compartmentalization, energy supply and metabolism, gene replication, biosynthesis, communication, growth and division, or motility.^6,8,15,18^ Materials must be engineered to optimize cytomimetic functions, including the nature of the charges, the multivalency and charge density of the polyelectrolytes, their charge ratio, or the presence of linkers and auxiliary monomers that tailor the polyelectrolyte chemical composition.^14,17^ Here, we demonstrate how dynamic covalent chemistry (DCvC) can significantly accelerate this lengthy and often behind-the-scenes process by activating polymers for *in situ* coacervation that would otherwise be inactive toward phase separation.

DCvC refers to reversible chemical transformations carried out under conditions of equilibrium control.^35–37^ One notable example is the formation of boronate esters from boronic acids (BA) and diols under aqueous conditions.^38–40^ Despite their widespread use in stimuli-responsive materials and drug delivery, BA have remained largely unexplored in the coacervate arena.^41^ Our approach exploits the simultaneous condensation of cationic and anionic catechols onto a dendritic BA to drive the formation of coacervate-based synthetic cells (Figure 1). Reminiscent of a polymeric gene delivery platform,^42^ the strategy yields dynamic zwitterionic polyboronates that phase separate into microdroplets, which are then stabilized as MCM via hierarchical assembly of a BA-functionalized dendritic block copolymer at their periphery. The robustness of this approach further entitles *in situ* adaptive optimization of the cytomimetic functions of MCM through dynamic covalent libraries, achieved by either tuning the charge ratio between oppositely charged catechols, adjusting the catechol-to-BA (CBA) ratio, or introducing auxiliary catechol dopants.

**Figure 1:**
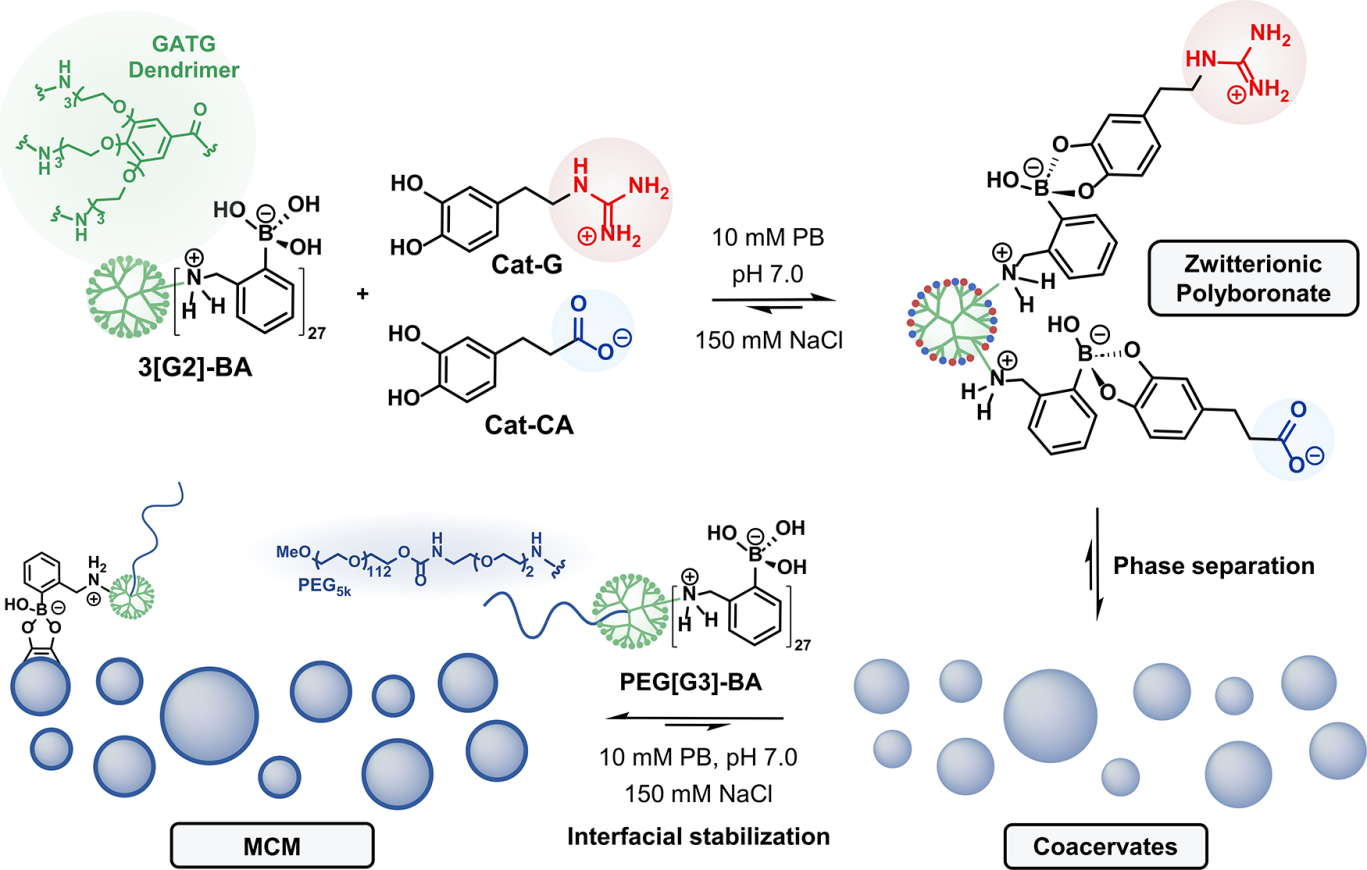
Schematic illustration of the *in situ* coacervation of a dendritic BA by the addition of oppositely charged catechols. Spontaneous phase separation of the resulting zwitterionic polyboronate, followed by interfacial stabilization with a BA-functionalized PEG-dendritic block copolymer, affords membranized coacervate microdroplets (MCM).

## Results and Discussion

### Dynamic Covalent Boronate Chemistry for the *In Situ* Formation and Interfacial Stabilization of Coacervates

To streamline the formation, interfacial stabilization, and biomimetic optimization of coacervates, we relied on the fast and strong boronate ester bond^38,39,42^ and a dendritic polymeric scaffold.^43,44^ Although dendrimers are ideal multivalent templates for new technologies and bioapplications,^45^ the advantages of their globular, tree-like, and rigid architecture in coacervate synthetic cells have only recently emerged.^28^ As earlier shown for nanosized, coacervate polyion complexes (PIC),^46–53^ the incorporation of charged dendrimers into MCM confers unprecedented stability in serum and under high ionic strength,^28^ overcoming a major challenge to the integrity of complex coacervates under physiological conditions.^54,55^ Fundamental differences in the local dynamics between linear polymers and dendrimers account for the increased stability. While the local dynamics of linear polymers are dictated by repeating segments that remain independent of molecular weight, dendrimers exhibit progressively slower internal dynamics toward the inner layers and with increasing generation.^56,57^

*In situ* phase separation was assessed through simultaneous conjugation of cationic and anionic catechols to 3­[G2]-BA, a dendrimer of the gallic acid-triethylene glycol (GATG)^53,54,58^ family bearing 27 peripheral BA groups (Figures 1 and SI). Since boronate ester formation is favored at pH values above the p*K*_a_ of the BA, 3­[G2]-BA incorporates *ortho*-aminomethylphenylboronic acids (p*K*_a_ ca. 6.5) to enable efficient ester bonding at physiological pH.^59^ 3­[G2]-BA does not coacervate in solution despite its zwitterionic nature at neutral pH (positive ammonium and negative BA groups).^60,61^ Conversely, upon simultaneous addition of equimolar amounts of oppositely charged carboxylated (Cat-CA) and guanidinylated (Cat-G) catechols (CBA ratio 1), the resulting dynamic polyboronate undergoes spontaneous *in situ* phase separation (Figure 1). The lifetime of this turbid coacervate suspension was nevertheless short, with droplets beginning to coalesce into a bulk phase after just 1 h, as confirmed by visual inspection and brightfield microscopy (Figure 2A). Long-term coacervate stability was achieved by interfacial stabilization with PEG­[G3]-BA, a PEG_5k_-dendritic block copolymer with 27 BA groups (PEG is polyethylene glycol) (Figure 1). When added to the suspension 30 min after the catechols, this copolymer assembles hierarchically on the surface of the coacervate microdroplets, leading to a stable dispersion of MCM with a mean diameter of 2.45 ± 0.63 μm by microscopy (Figure 2A). Successful stabilization was demonstrated by droplet lifetimes extending beyond 1 week as assessed by brightfield microscopy and turbidity analysis (Figures 2A and S2). Interfacial stabilization studies revealed an optimal PEG­[G3]-BA loading corresponding to 9 mol % of 3­[G2]-BA. Lower concentrations produced a marked decrease in long-term turbidity, indicating a less efficient stabilization (Figure S3). The *z*-potential of the MCM was close to zero (Figure S1), consistent with the presence of a PEGylated membrane. As anticipated for systems built from a fully dendritic framework,^51^ polyboronate MCM exhibit exceptional salt resistance, outperforming coacervates derived from arginine-rich peptides.^62^ No size variation or disintegration of the droplets was observed by microscopy as the NaCl concentration increased up to 4 M, with turbidity remaining constant throughout (Figure 2B).

**Figure 2:**
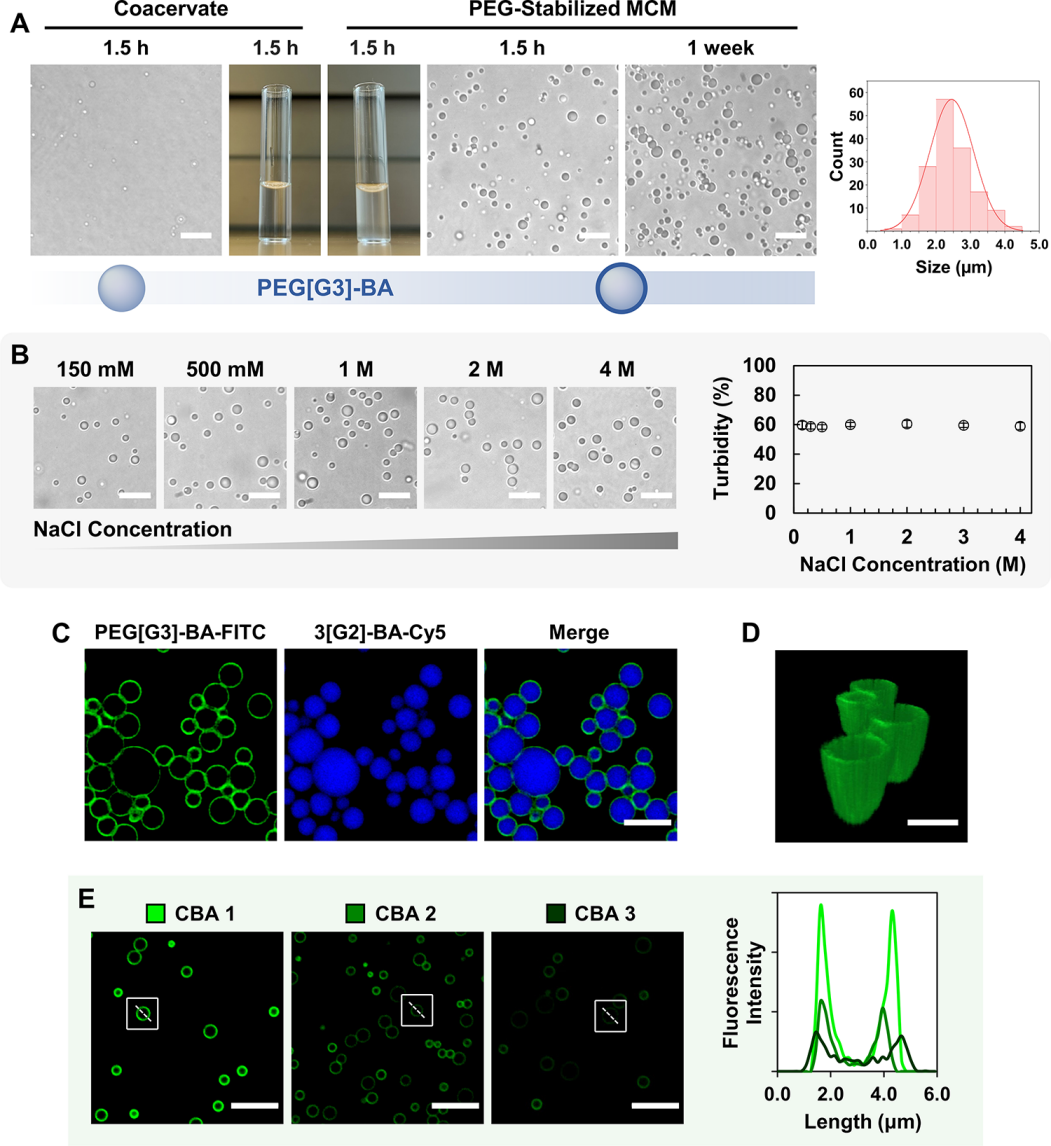
Interfacial stabilization of polyboronate coacervates with PEG­[G3]-BA provides MCM with long-term and ionic strength stability. Scale bars: 10 μm. Histogram showing the MCM size distribution (180 droplets analyzed) (A, B). CLSM images of double fluorescently labeled MCM show PEG­[G3]-BA-FITC (green) hierarchically assembled at the external interface and 3­[G2]-BA-Cy5 (blue) confined at the core of the droplets. Scale bar: 5 μm (C). 3D reconstructed image showing selective peripheral localization of PEG­[G3]-BA-FITC. Scale bar 2 μm (D). CLSM images of MCM prepared with different CBA ratios show a more efficient membranization with PEG­[G3]-BA-FITC (green) at CBA 1. Cross-sectional fluorescence intensity profiles of selected droplets. Scale bars 10 μm (E).

Evidence of boronate ester formation within the coacervate and its dynamic nature was obtained by using ^1^H NMR spectroscopy (Figure 3). Treatment of 3­[G2]-BA with Cat-CA led to the immediate disappearance of characteristic 3­[G2]-BA signals centered at 7.36 (H_1BA_) and 3.02 ppm (H_2BA_) and the appearance of new ester peaks at 7.55 (H_1Cat‑CA_) and 3.11 ppm (H_2Cat‑CA_). A 70% conversion was determined by the relative integration of the H_1_ and H_2_ protons. A similar treatment with caffeic acid, a higher-affinity catechol, afforded a 95% conversion. It is noteworthy that relatively higher conversions than those observed in solution are expected at CBA 1 within the MCM. The crowded local coacervate environment selectively enhances the effective concentration of reagents (3­[G2]-BA and catechols).^63^ Furthermore, while the overall water content of coacervates is only slightly reduced compared to the external milieu,^63^ the amount of “free” bulk water—not bound within hydration shells (“structured” water)—is significantly lower.^64^ This leads to a reduced effective water reactivity that favors condensation reactions.^63,65^ Competitive ^1^H NMR experiments were subsequently conducted. Cross-addition of caffeic acid and Cat-CA (CBA 6) to their respective preformed Cat-CA and caffeic acid boronate esters (samples A and B in Figure 3) produced the immediate and quantitative formation of an identical mixed boronate ester (1:9 Cat-CA:caffeic acid ratio), confirming the dynamic nature of the boronate ester bond.

**Figure 3:**
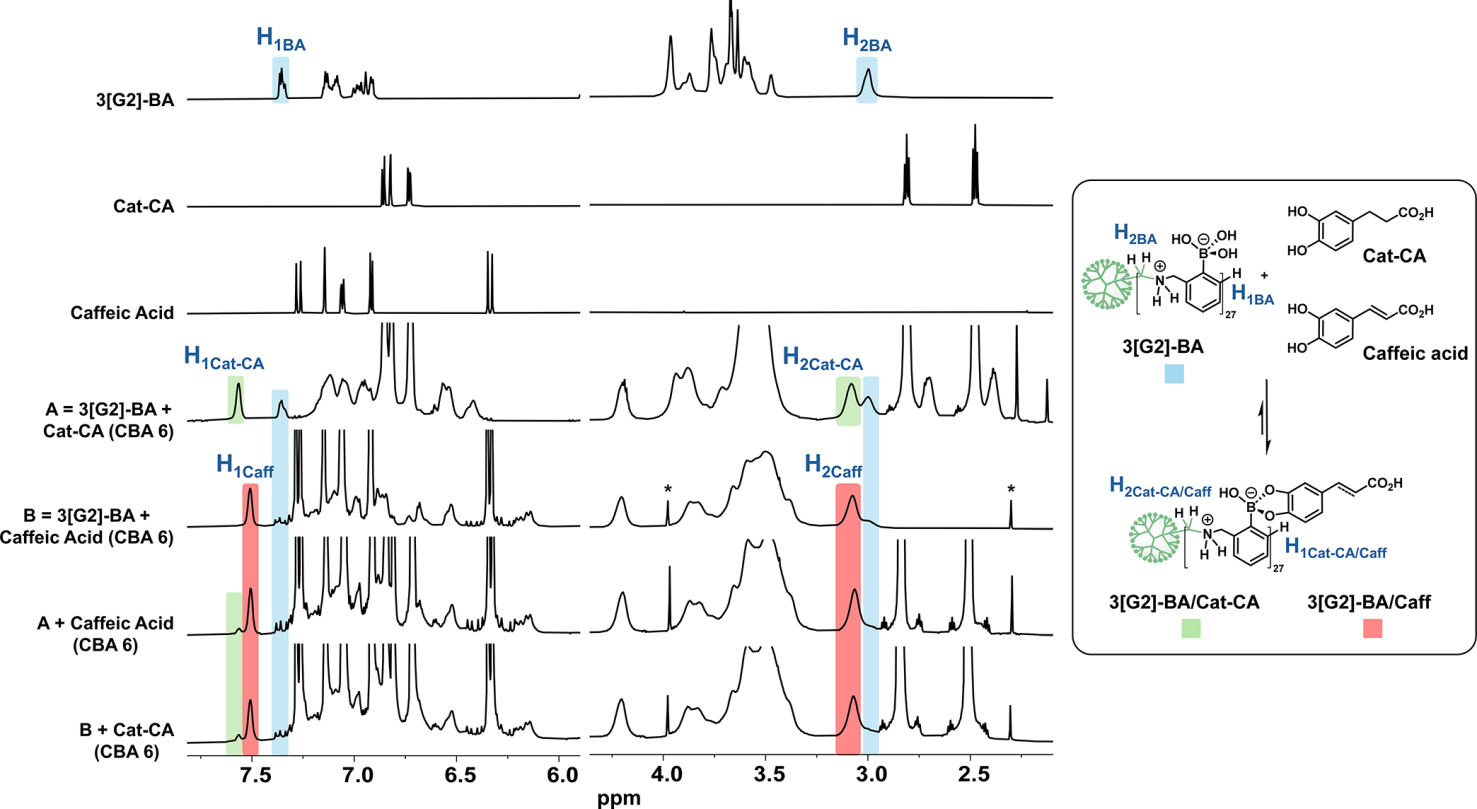
Dynamic covalent nature of the boronate ester bond by ^1^H NMR (750 MHz, D_2_O, pH 7.0). Cat-CA and caffeic acid, a higher-affinity catechol competitor, were added (CBA 6) to 3­[G2]-BA (A, B). Boronate ester formation was confirmed by the disappearance of 3­[G2]-BA signals (H_1BA_ and H_2BA_) and the appearance of new peaks (H_1Cat‑CA_, H_2Cat‑CA_, H_1Caff_, and H_2Caff_). Relative integration of H_1_ and H_2_ protons revealed 70% conversion for Cat-CA and 95% conversion for caffeic acid. Cross-addition of caffeic acid and Cat-CA (CBA 6) to (A, B) consistently led to the immediate and quantitative formation of the same mixed boronate ester (1:9 Cat-CA/caffeic acid ratio).

Selective membranization of the MCM was confirmed by confocal laser scanning microscopy (CLSM) using a double fluorescently labeled version of the MCM prepared using PEG­[G3]-BA-FITC and 3­[G2]-BA-Cy5, which incorporate fluorescein isothiocyanate (green) and Cyanine 5 (blue), respectively. Figure 2C,D shows a well-defined MCM organization with a green coating membrane and a blue interior, indicating that PEG chains from PEG­[G3]-BA are selectively exposed at the external interface while 3­[G2]-BA is confined within the droplets’ interior. The influence of the CBA ratio on the efficiency of coacervation and membranization was then analyzed. CLSM analysis of MCM prepared with different CBA ratios (1, 2, and 3; equimolecular charge ratio of Cat-CA and Cat-G) revealed fully stable MCM with a green fluorescent membrane (FITC), confirming successful copolymer assembly at the droplet surface for all three CBA ratios. Nevertheless, significant differences in fluorescence intensity were observed across the samples. Specifically, CBA ratios 2 and 3 showed intensities markedly lower than those of CBA 1 (Figure 2E). This reduction is attributed to the unproductive esterification of PEG­[G3]-BA with excess catechols in the external milieu, preventing effective membranization at higher CBA ratios. Consistent with this interpretation, Figure S5 shows that PEG­[G3]-BA assembles into nanometer-sized micelles when excess Cat-CA and Cat-G are present. These results were confirmed by quantifying the fluorescence of unbound PEG­[G3]-BA-FITC in the diluted phase after centrifugation. Membranization efficiencies of 72% PEG­[G3]-BA for CBA 1, 63% for CBA 2, and 43% for CBA 3 were obtained (Figure S6). Interestingly, CLSM imaging showed that coacervates (CBA 1) stabilized with the copolymer preassembled into micelles had even lower fluorescence intensity membranes than CBA 3, with a corresponding membranization efficiency of 32% (Figures S6 and S7). These results confirm that individual copolymer chains, rather than preformed micelles, are responsible for coacervate membranization. Accordingly, a CBA ratio of 1 was set for all subsequent experiments.

### Cytomimetic Functions of Polyboronate MCM: Encapsulation of Biological Macromolecules and Chemical Communication

The capacity of polyboronate MCM to emulate life-like behavior, such as recruitment of biomacromolecules and chemical communication between caged populations, was assessed using the enzymatic cascade system composed of glucose oxidase (GOX, 160 kDa, pI 4.2; pI is the isoelectric point) and horseradish peroxidase (HRP, 44 kDa, pI 9.0). Both enzymes were fluorescently labeled with Cy5 and encapsulated into distinct MCM populations by adding them immediately after the addition of 3­[G2]-BA with Cat-CA and Cat-G.

Optimization of enzyme partitioning in coacervates is typically addressed by modulating the affinity of the cargo for the inner core of the coacervates. While numerous studies depend on protein modifications to enhance entrapment,^66,67^ alternative approaches involve using one polyelectrolyte in excess.^68^ Here, the robustness of DCvC enabled an adaptation of this latter strategy to enhance protein partitioning within the coacervates by tuning the molar ratio between charged catechols, which allowed for modulation of the droplet inner environment without the need to modify the polymeric scaffold. Three different Cat-CA/Cat-G charge ratios were tested (2:1, 1:1, and 1:2), showing no significant effect on the size, stability, and *z*-potential of the MCM (Figures 4 and S1). For the three charge ratios, the encapsulation efficiency (EE) of GOX and HRP remained high, regardless of their molecular weight and pI. Nonetheless, slightly larger protein partitioning was observed with increasing proportions of Cat-G: EE increasing from 83 to 96% for GOX and from 52 to 71% for HRP (Figure 4). This effect, likely arising from the hydrogen bonding capacity of the guanidinium group, underscores the potential of dynamic covalent libraries for the adaptive optimization of biomimetic functions in synthetic cells. As shown in Figure 4C, these encapsulation conditions proved broadly applicable to other proteins, including antibodies, as well as a plasmid DNA, with high EE values: lysozyme (14 kDa, pI 11.4; EE 71%), bovine serum albumin (BSA, 66 kDa, pI 4.7; EE 88%), insulin (6 kDa, pI 5.3; EE 92%), immunoglobulin G (IgG, 150 kDa; EE 55%), and pEGFP-N1 (4733 bp; EE 100%). These high EE establish polyboronate MCM as a versatile biomimetic platform for the efficient encapsulation of diverse biomolecules (irrespective of their size and charge), thereby offering promising avenues for the design of advanced delivery and compartmentalization systems. Subsequent enzymatic cascade experiments were performed using MCM prepared with a 1:2 Cat-CA/Cat-G ratio.

**Figure 4:**
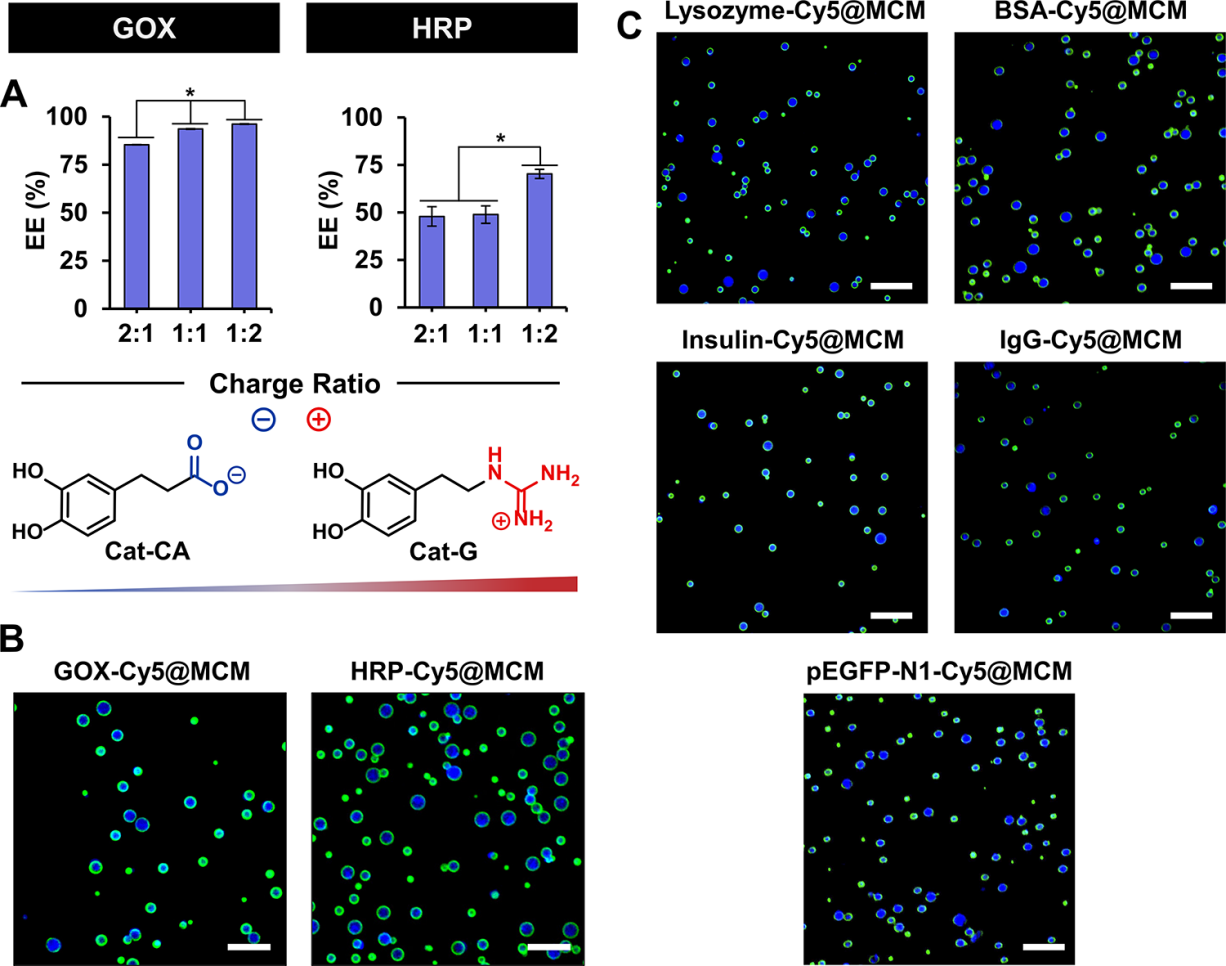
Protein encapsulation efficiency (EE) into MCM prepared with varying Cat-CA/Cat-G charge ratios (CBA 1). (*) indicates statistical difference (*p* < 0.05), analyzed by one-way ANOVA, followed by a Tukey multiple comparisons test (A). CLSM images of MCM (Cat-CA/Cat-G 1:2, CBA 1) interfacially stabilized with PEG­[G3]-BA-FITC (green) and loaded with Cy5-fluorescently labeled GOX, HRP (B), lysozyme, BSA, insulin, anti-BSA rabbit IgG, and a pDNA (pEGFP-N1) (C). Cy5 is shown in blue. Scale bars 10 μm.

The GOX-HRP enzymatic cascade involves the oxidation of β-d-glucose by GOX in the presence of O_2_ to produce d-glucono-1,5-lactone and H_2_O_2_, which is subsequently used by HRP to catalyze a second oxidation reaction. The progress of the enzymatic cascade can be followed by confocal microscopy or fluorescence spectroscopy using Amplex Red or *o*-phenylenediamine (oPD) as nonfluorescent HRP substrates, which are oxidized to the fluorescent products resorufin and 2,3-diaminophenazine (2,3-DAP), respectively (Figure 5). Since maintaining active droplet–droplet communication after interfacial stabilization represents a challenge in synthetic biology, the incorporation of GOX and HRP in distinct MCM populations (Cat-CA/Cat-G 1:2, CBA 1) was envisaged to assess their membrane permeability for small substrates (glucose, Amplex Red, and oPD) and chemical signals (H_2_O_2_) as well as their ability to function as independent compartments. The efficiency of the enzymatic cascade was initially investigated by CLSM experiments following the appearance of the fluorescence signal of resorufin (red) after the addition of glucose to a mixture of Amplex Red and independent MCM populations loaded with fluorescently labeled versions of the enzymes: GOX-AF488 (green) and HRP-Cy5 (blue) (Figure 5A). The visualization of the resorufin fluorescence in the MCM population loaded with HRP-Cy5 after only 1 min of reaction time confirmed fast enzymatic reactions and communication between droplets. After 10 min, red fluorescence was also detected within the GOX-AF488 compartments due to resorufin equilibration between MCM populations as earlier described.^25,53^ Notably, the semipermeable membrane allows the diffusion of small molecules but retains macromolecular enzymes effectively, as protein exchange was undetectable even after 3 h. Control experiments carried out under identical conditions in the absence of either GOX- or HRP-loaded MCM did not produce resorufin fluorescence, confirming the necessity of both enzyme-loaded synthetic cells for a successful enzymatic cascade (Figure S9).

**Figure 5:**
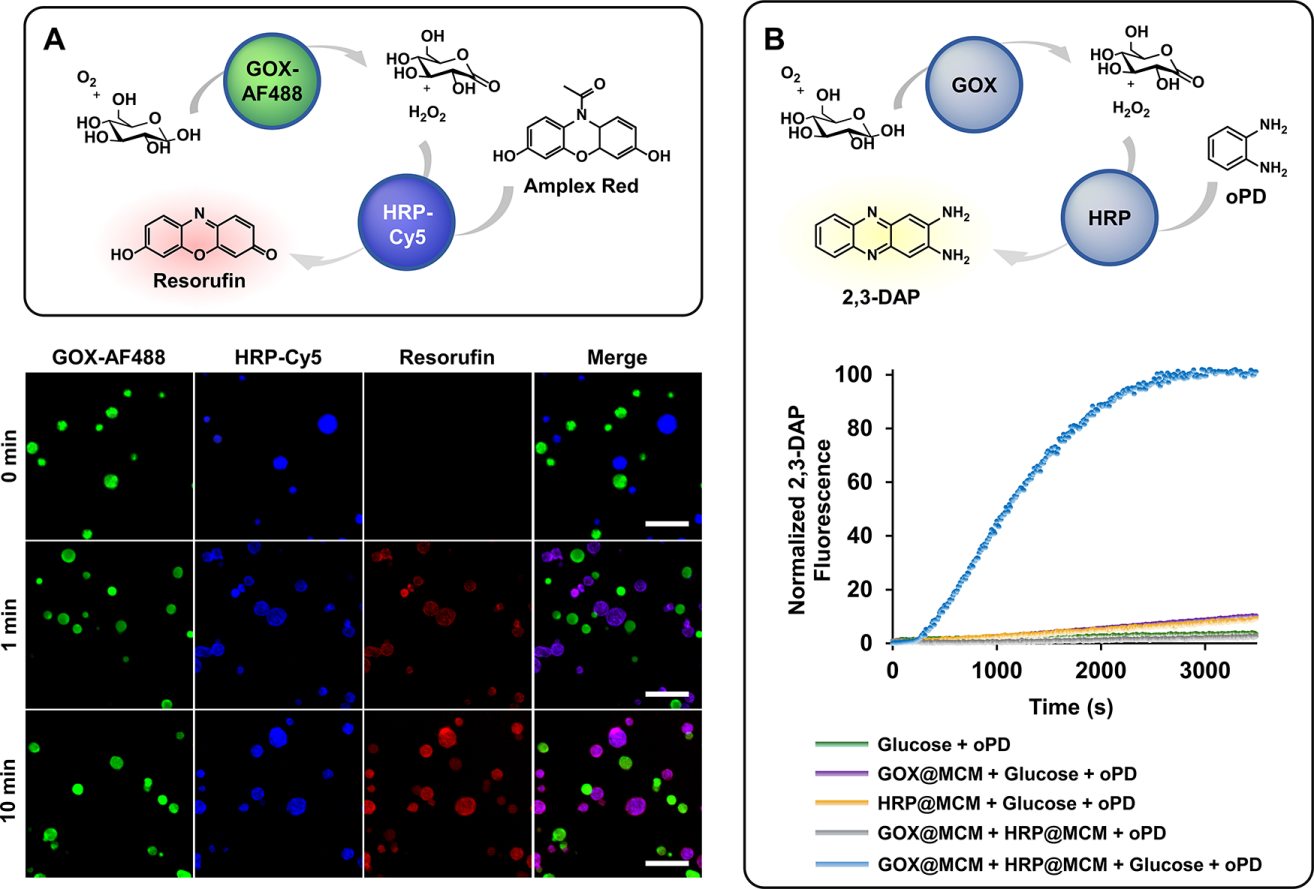
Scheme of the GOX-HRP enzymatic cascade and the chemical communication between MCM populations. CLSM images of the enzymatic production of resorufin (red) from GOX-AF488@MCM (green) and HRP-Cy5@MCM (blue) in the presence of Amplex Red before (0 min) and after (1 and 10 min) addition of glucose. Scale bars 10 μm. (A). Progress of the enzymatic cascade between GOX@MCM and HRP@MCM was studied using oPD as the HRP substrate by monitoring the fluorescence of the reaction product 2,3-DAP (B).

To analyze the time-dependence of the enzymatic cascade, oPD was chosen as HRP substrate instead of Amplex Red (Figure 5B), which is unsuitable for such experiments because it undergoes photooxidation to resorufin upon continuous exposure to light, a process initiated by trace amounts of resorufin inherently present in the reagent.^69^ Continuous measurement of the 2,3-DAP signal by fluorescence spectroscopy was used to monitor the progress of the enzymatic cascade between GOX@MCM and HRP@MCM in the presence of glucose and oPD (65 nM GOX and 85 nM HRP). Less than 1 h after the addition of glucose to initiate the reaction, a maximum of 2,3-DAP fluorescence was reached, indicating completion of the process (Figure 5B). As described above, control experiments showed no fluorescence in the absence of any of the enzymes or glucose (Figures 5B and S10). Overall, these findings validate the ability of polyboronate MCM to emulate cell-like behavior such as enzyme encapsulation and chemical communication, while preventing macromolecular migration between droplets.

### Cytomimetic Functions of Polyboronate MCM: Permeability of the PEG-Dendritic Copolymer Membrane

Membranization endows MCM with essential cytomimetic properties, stabilizing them against coalescence while selectively regulating the uptake and exclusion of extracellular species, much like natural cell membranes.^34^ The permeability of MCM membranes is mainly governed by membrane fluidity and dynamics, with larger blocks increasing porosity.^34^ For example, while MCM enclosed within a phospholipid membrane excludes a 4 kDa dextran,^23^ interfacial stabilization with PEGylated Au nanoparticles,^29^ metal-phenolic networks,^30^ a terpolymer-based membrane,^25,66^ or a multi-PEGylated BSA conjugate^70^ enables its efficient uptake. Motivated by the rapid equilibration of small molecules but the absence of protein exchange between coacervate populations seen in Figure 5, a CLSM study was performed to assess the permeability of the PEG-dendritic membrane to species of different charge and size: methylene blue (cationic, 320 Da), pyranine (anionic, 524 Da), propidium iodide (cationic, 668 Da), lysozyme-Cy5 (cationic, 14 kDa), HRP-Cy5 (cationic, 44 kDa), and GOX-Cy5 (anionic, 160 kDa). Upon incubation with solutions of the small dyes and globular proteins, MCM showed a pronounced increase in internal fluorescence for all three dyes and for lysozyme. In contrast, the fluorescence increase was modest for HRP and decreased further for GOX, which exhibited only minimal uptake in a small fraction of the MCM population (Figures 6A and S11). This observation reflects a progressively reduced permeability toward higher molecular weight proteins and an independence of charge effects. Interestingly, partition coefficients obtained from the concentrations of the guests in the coacervate and dilute phases (Figure 6B) confirmed the size-dependent trend observed by CLSM. Given the reported hydrodynamic diameters of lysozyme (4 nm),^71^ HRP (7–8 nm),^72,73^ and GOX (10–12 nm),^74,75^ the uptake profile suggests that the PEG-dendritic membrane functions as a semipermeable barrier with an apparent pore size of approximately 10–12 nm. To enable controllable transport in MCM, recent studies have turned to engineering membranes with programmed^76^ or stimuli-responsive permeability.^29^ In this context, the recently reported link between the PEG block length in PEG-dendritic block copolymers and their membrane packing density in coacervate PIC micelles^52^—shorter PEG chains yield more densely PEGylated membranes—provides an opportunity to tune MCM permeability.

**Figure 6:**
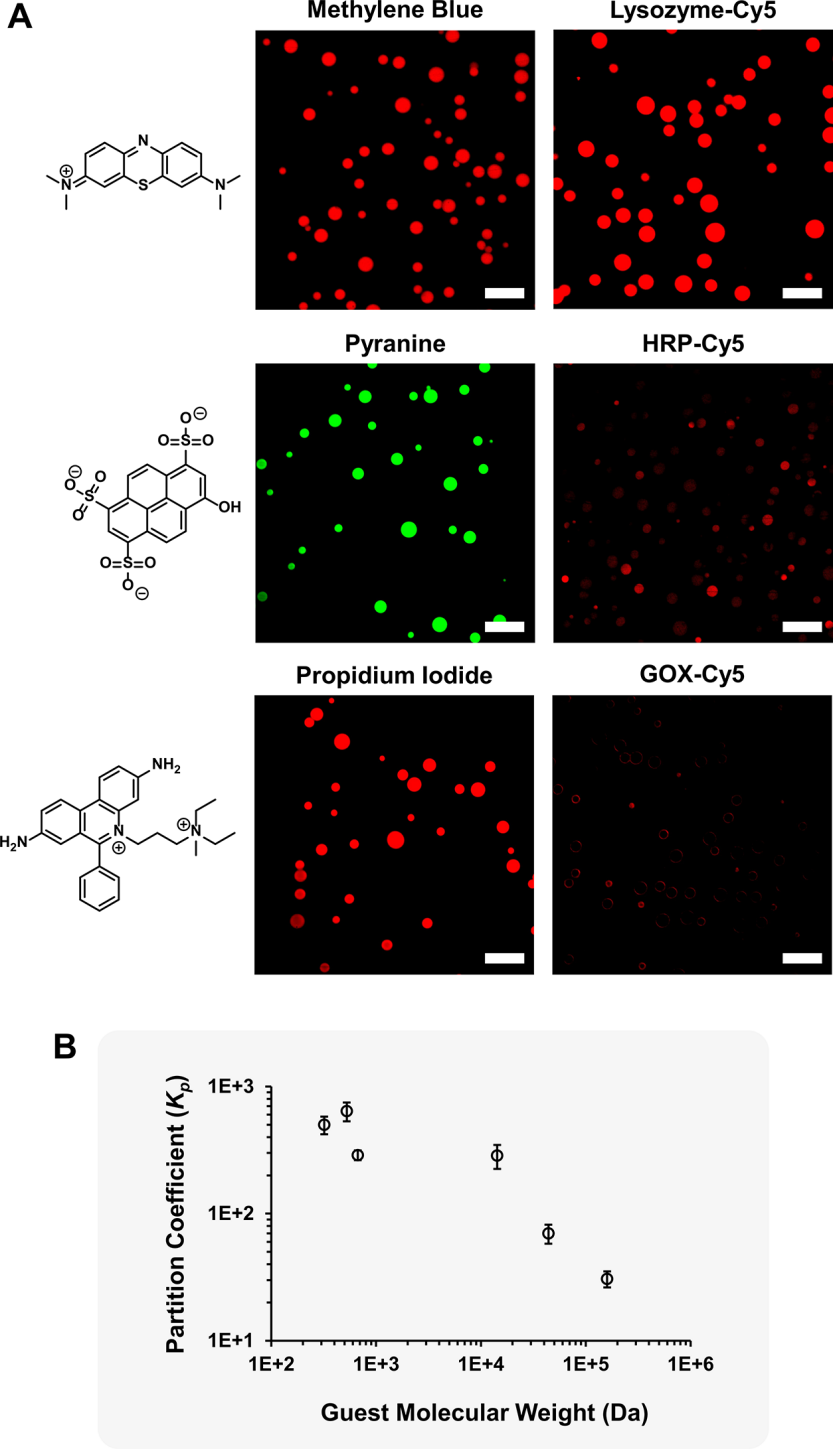
Permeability of the MCM membrane (Cat-CA/Cat-G 1:1, CBA 1) to different guests assessed by CLSM (10 min of equilibration). Scale bars: 10 μm. (A) Partition coefficients (*K*_p_) (B). Guests (net charge at pH 7.0, molecular weight): methylene blue (cationic, 320 Da), pyranine (anionic, 524 Da), propidium iodide (cationic, 668 Da), lysozyme-Cy5 (cationic, 14 kDa), HRP-Cy5 (cationic, 44 kDa), and GOX-Cy5 (anionic, 160 kDa).

### Optimizing Cytomimetic Functions of Polyboronate MCM by Dynamic Covalent Libraries

Recent reports have shown that variations in the multivalency, chemical nature, and charge density of polyelectrolytes undergoing phase separation significantly influence the internal dynamics and enzymatic activity of complex coacervates.^77,78^ As in natural cells, where activity and viscosity are intrinsically linked,^79^ enzymatic function in coacervate synthetic cells also requires effective diffusion. While the classical approach of such structure–activity endeavors involves synthesizing a series of polyelectrolytes with specific structural variations, boronate chemistry enables the adaptive optimization of cell-like behavior through dynamic covalent libraries to modulate the coacervate inner environment *in situ*. This eliminates the need to synthesize, isolate, purify, and characterize optimized polyelectrolyte materials. To explore this approach, epicatechin (EPI), epigallocatechin gallate (EGCG), and catechol (CAT) were assessed as uncharged dopants to enhance the internal dynamics, enzymatic activity, and chemical communication among polyboronate MCM (Figure 7). Doped MCM (10 mol % catechol dopant relative to BA, Cat-CA/Cat-G 1:2, CBA 1), interfacially stabilized with PEG­[G3]-BA-FITC, were prepared and analyzed using brightfield and CLSM. The resulting droplets were indistinguishable from those of undoped MCM (Figure S8).

**Figure 7:**
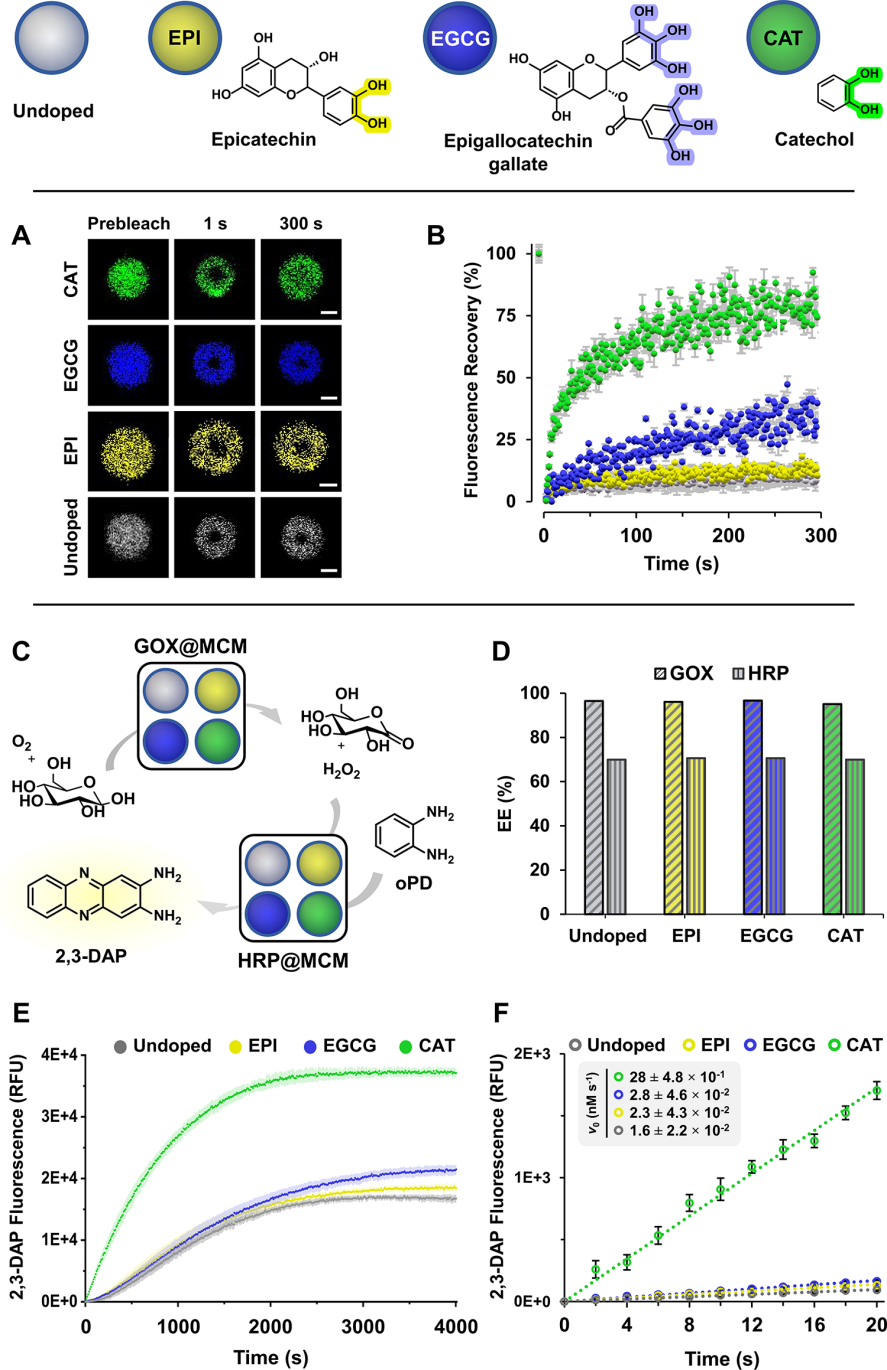
Optimizing the enzymatic activity and chemical communication of polyboronate MCM (undoped control, gray) by doping with epicatechin (EPI, yellow), epigallocatechin gallate (EGCG, blue), and catechol (CAT, green). Images of fluorescence recovery after photobleaching (FRAP) experiments (A, scale bars 1 μm) and fluorescence recovery plots versus time of 3­[G2]-BA-Cy5 in undoped and doped MCM (B). Enzymatic cascade and chemical communication between GOX@MCM and HRP@MCM (undoped and doped) using oPD as the HRP substrate (C). Encapsulation efficiency (EE) of GOX and HRP in doped MCM (D). Progress of the enzymatic cascade in undoped and doped MCM was studied by monitoring the fluorescence intensity of the reaction product 2,3-DAP (E). Linearized plots of 2,3-DAP fluorescence versus time reveal a 17.5-fold increase in reaction kinetics when doping MCM with CAT. *R* ^2^ ≥ 0.996 in all cases (F).

The impact of doping on the phase fluidity and internal mobility of coacervates was examined by fluorescence recovery after photobleaching (FRAP) experiments using 3­[G2]-BA-Cy5 (Figure 7A). Monitoring fluorescence recovery in a photobleached region, caused by the internal mobility of neighboring dendrimers, provides information on their diffusion rate within the cell (see the SI).^80^ Figure 7B shows the fluorescence recovery for 3­[G2]-BA-Cy5 in undoped and doped MCM. The doped systems exhibited markedly higher recovery rates, indicating enhanced diffusion and internal fluidity. This was particularly evident for the smaller CAT dopant, for which an apparent diffusion coefficient (*D*_app_) of 1.39·10^–3^ ± 7.89·10^–5^ μm^2^/s was determined by fitting the FRAP recovery curve to an exponential function (Figure S12).

The effect of this increased fluidity on enzymatic activity and chemical communication between MCM was evaluated by using the GOX-HRP enzymatic cascade (Figure 7C). Independent populations of MCM, doped with EPI, EGCG, and CAT and encapsulating GOX and HRP, were prepared with EE comparable to their undoped counterparts (Figure 7D). The enzymatic cascade was analyzed using oPD as the HRP substrate by monitoring the fluorescence of the 2,3-DAP product (65 nM GOX and 85 nM HRP). Doped MCM showed faster 2,3-DAP production in the order CAT ≫ EGCG > EPI > undoped (Figure 7E), consistent with the internal dynamics revealed by FRAP. Although end point fluorescence intensities in Figure 7E varied among MCM, these differences did not reflect a disparity in reaction extent. Thus, upon MCM disassembly with 1.6 M urea in MeOH (as confirmed by brightfield, Figure S13B), fluorescence intensities leveled off across all samples (Figure S13C), pointing to a dopant-dependent quenching of the 2,3-DAP fluorescence within the coacervate compartments, which was more pronounced for MCM with slower FRAP dynamics. To quantify the effect of doping on reaction kinetics, initial reaction rates (*v*_0_) were determined by linear fitting of time-dependent 2,3-DAP fluorescence over the first 20 s of the reaction, a time scale free of quenching (see the SI). Increasing *v*_0_ values from 1.6 ± 2.2 × 10^–2^ nM s^–1^ in the undoped system up to 2.3 ± 4.3 × 10^–2^ (EPI), 2.8 ± 4.6 × 10^–2^ (EGCG), and 28 ± 4.8 × 10^–1^ nM s^–1^ (CAT) in the doped MCM were obtained (Figures 7F and S15), corresponding to a 17.5-fold enhancement in reaction kinetics. This result, consistent with recent reports linking reduced charge density in polyelectrolytes to increased internal mobility and enhanced enzyme and ribozyme activity within complex coacervates,^77,78^ highlights the potential of dynamic covalent boronate chemistry to adaptively optimize cytomimetic functions of coacervates using dynamic covalent libraries.

Finally, given the promising recent advances in synthetic-natural cell integration,^28,81–84^ the pH-dependent stability and cytotoxicity of MCM were evaluated to determine their suitability for such applications. The pH-sensitivity of the boronate ester bond^59^ provides a valuable mechanism to regulate the stability and function of polyboronate MCM across diverse biological environments, reflecting the wide range of pH conditions (i.e., pH 7.4 for healthy tissue, 6.8 for tumor microenvironment, 5.0–6.5 for endosomes, and 4.5–5.0 for lysosomes). MCM stability was monitored over 24 h by measuring turbidity under increasingly acidic conditions (Figure S4). Whereas the droplets remained fully stable at pH 7.4, a progressive decrease in turbidity was observed as the pH was lowered, leading to complete coacervate disassembly at pH 4.0, a finding consistent with the pH-responsive nature of the boronate ester bond. The cytotoxicity of MCM was evaluated in A549 cells using the CCK-8 assay. Cell proliferation was only marginally affected, even after 72 h at the highest concentrations analyzed, with viabilities consistently exceeding 90% (Figure S17). In addition, microscopy experiments demonstrated that MCM maintains membrane integrity and stable size in Dulbecco’s modified Eagle’s medium (DMEM) with high glucose (a competitor of catechol for the BA groups in the dendrimer), containing 10% fetal bovine serum (Figure S16). Overall, these results pave the way for MCM applications in advanced therapies, tissue engineering, and regenerative medicine.^85–88^

## Conclusions

Although significant progress has been achieved in modeling cell-like behavior using synthetic cells, material engineering remains a bottleneck in the optimization of biomimetic properties. Here, we describe how dynamic covalent chemistry can streamline this process. The potential of dynamic covalent boronate chemistry for the *in situ* formation, interfacial stabilization, and adaptive cytomimetic optimization of membranized coacervate microdroplets (MCM) is shown. Simultaneous addition of cationic and anionic catechols to a dendritic boronic acid (BA) generates dynamic zwitterionic polyboronates that phase separate into microdroplets that can be interfacially stabilized with a BA-functionalized block copolymer. The cytomimetic properties, membranization, internal dynamics, and enzymatic activity within the MCM can be modulated using dynamic covalent libraries by tuning the charge ratio between oppositely charged catechols, adjusting the catechol-to-BA (CBA) ratio, or introducing auxiliary catechol dopants that fine-tune material properties. Notably, this technology avoids the need to synthesize, isolate, purify, and characterize polymeric materials, accelerating the optimization of cytomimetic behavior and functions. Given the repertoire of intermolecular interactions driving the formation of coacervates (electrostatic, dipolar, hydrogen-bond, cation−π, π–π, biomolecular recognition),^14,17^ extending the range of catechols to include hydrophobic, polar, and other charged variants encoding multiple interactions is anticipated to broaden the applicability and versatility of the strategy. In addition, catechols bearing stimuli-responsive functionalities are also envisaged to provide programmable polyboronate MCM with emerging properties. Application of the technology to other polymeric BA and synthetic cell architectures holds promise for the optimization of diverse biomimetic functions.

## Supplementary Material



## Notes

Materials, instrumentation, experimental procedures and characterization of membranized coacervate microdroplets, enzymatic cascade assays, and cell viability studies (PDF)
