# Supplementary material for: Dynamic Covalent Boronate Chemistry for In Situ Formation, Interfacial Stabilization, and Cytomimetic Optimization of Coacervates
Source: J Am Chem Soc. 2026 Feb 27;148(9):9346–57. doi: 10.1021/jacs.5c17688 (PMC12983306; doi:10.1021/jacs.5c17688)
Supplement: Supplementary file 1 [file ja5c17688_si_001.pdf]

Supporting Information

Dynamic Covalent Boronate Chemistry for *In Situ* Formation, Interfacial Stabilization, and Cytomimetic Optimization of Coacervates

Bruno Delgado Gonzalez,[§] *Lucas Garcia-Abuin,*[§] *Celia Jimenez-Lopez, and Eduardo Fernandez-Megia**

Centro Singular de Investigación en Química Biolóxica e Materiais Moleculares (CIQUS), Departamento de Química Orgánica, Universidade de Santiago de Compostela, Jenaro de la Fuente s/n, 15782 Santiago de Compostela, Spain

Table of Contents

1. Materials S3

2. Instrumentation S3

3. Preparation of Fluorescently Labeled Compounds S5

4. Preparation and Characterization of Membranized Coacervate Microdroplets (MCM) S8

5. Enzymatic Cascade Assays S21

6. Permeability of the MCM Membrane S25

7. Optimizing Cytomimetic Functions of MCM by Dynamic Covalent Libraries S29

8. Cell Viability Studies S36

9. References S38

1. Materials

N-Acetyl-3,7-dihydroxyphenoxazine (Amplex Red) was supplied by Biosynth. AF488-NHS and Cy5-NHS were obtained from Lumiprobe. Cell Counting Kit-8 (CCK-8) was purchased from TargetMoi. Glucose oxidase (GOX) from *Aspergillus niger*, peroxidase from horseradish type VI (HRP), bovine serum albumin fraction V (BSA), human recombinant insulin, and lysozyme from chicken egg white were supplied by Sigma-Aldrich. Anti-BSA rabbit IgG was purchased from Invitrogen. The production of a plasmid DNA (pEGFP-N1, 4733 bp) and its fluorescent labeling with Cy5 were done following previous procedures reported by our group.[1] Cat-G was prepared following known procedures.[2] PEG[G3]-BA and 3[G2]-BA were prepared following procedures previously described by our group.[1] All other chemicals were purchased from Acros Organics, Fluka, Sigma-Aldrich, or Thermo Fisher Scientific, unless otherwise noted. All solvents were HPLC grade, purchased from Scharlab or Sigma-Aldrich. CH_2Cl_2 was dried using a SPS800 solvent purification system from MBRAUN. DMSO and Et_3N were dried under 4Å molecular sieves. H_2O was of Milli-Q grade obtained using a Millipore water purification system.

2. Instrumentation

Determination of pH Values. pH values were measured with a portable pH-meter (Crison PH25) connected to a glass electrode (Crison 52 09).

Dialysis. Dialysis was performed with an 18 mm Spectra/Por 6 (MWCO 1 or 10 kDa) membrane tubing from SpectrumLabs.

UV-Vis Spectroscopy. UV-Vis spectra were recorded on a Jasco V-630 spectrophotometer or a plate reader Tecan Infinite F200 PRO.

Zeta Potential. Z-potential values were measured by laser doppler anemometry (LDA), measuring the mean electrophoretic mobility (Malvern Zetasizer Nano ZS, Malvern Instruments) in 10 mM PB pH 7.0, 150 mM NaCl using the Smoluchowski approximation.

Fluorescence Spectroscopy. Fluorescence measurements were performed in a plate reader Tecan Infinite F200 PRO.

Confocal Laser Scanning Microscopy (CLSM). Confocal images were obtained on an Andor Dragonfly spinning disk confocal system mounted on a Nikon TiE microscope equipped with a Zyla 4.2 PLUS sCMOS digital camera (Andor, Oxford Instruments). Samples were excited with different lasers (405, 488, 561, and 637 nm) and the emitted fluorescence was collected by the filter wheel (525/50 nm, 620/50 nm, and 725/40 nm) with appropriate combinations of them. Images were taken with 100× magnification objective. Images were processed using ImageJ software (version 1.51j8) and Imaris Viewer software (version 10.2.0).

The individual channels were recorded sequentially using the following parameters:

- Pyranine: excitation at 405 nm; emission at 525/50 nm.
- FITC & AF488: excitation at 488 nm; emission at 525/50 nm.
- Resorufin & propidium iodide: excitation at 561 nm; emission at 620/50 nm.
- Cy5 & methylene blue: excitation at 637 nm; emission at 725/40 nm.

Fluorescence Recovery After Photobleaching (FRAP). FRAP experiments were performed on an inverted CLSM Leica Stellaris 8 FALCON (Leica Microsystems, Wetzlar, Germany) employing the FRAP interface available in Leica Application Suite X (LAS X) software.

3. Preparation of Fluorescently Labeled Compounds

PEG[G3]-BA-FITC. A solution of fluorescein 5(6)-isothiocyanate (FITC, 0.90 mg, 2.40 µmol) in dry DMSO (188 µL) was added to a solution of PEG[G3]-BA (5.00 mg, 0.30 µmol) and Et_3N (0.2 µL, 1.21 µmol) in CH_2Cl_2 (262 µL). After stirring overnight at rt under Ar protected from light, the solvent was evaporated. The crude product was dissolved in 0.1 M $NaHCO_3$ and purified by dialysis (2 × 1.5 L 0.1 M $NaHCO_3$, 2 × 1.5 L H_2O; Spectra/Por 6, MWCO 1 kDa). After freeze-drying, PEG[G3]-BA-FITC (4.40 mg, 86%) was obtained as an orange solid. A degree of functionalization of 25% in fluorescein was determined by absorbance at 490 nm (ε_{490}: 73000 $cm^{-1}M^{-1}$ as provided by supplier).

3[G2]-BA-Cy5. A solution of Cy5-NHS (4.81 mg, 7.20 µmol) in dry DMSO (564 µL) was added to a solution of 3[G2]-BA (10.00 mg, 0.90 µmol) and Et_3N (0.6 µL, 3.601 µmol) in CH_2Cl_2 (786 µL). After stirring overnight at rt under Ar protected from light, the solvent was evaporated. The crude product was dissolved in 0.1 M $NaHCO_3$ and purified by dialysis (2 × 1.5 L 0.1 M $NaHCO_3$, 2 × 1.5 L H_2O; Spectra/Por 6, MWCO 1 kDa). After freeze-drying, 3[G2]-BA-Cy5 (9.18 mg, 88%) was obtained as a blue solid. A degree of functionalization of 9% in Cy5 was determined by absorbance at 646 nm (ε_{646}: 250000 $cm^{-1}M^{-1}$ as provided by supplier).

General Procedure for Fluorescent Labeling of Proteins. Proteins were dissolved at 5 mg/mL in 0.1 M $NaHCO_3$ pH 9.0. A fresh solution of the fluorescent dye in DMSO was added and the reaction mixture was stirred overnight at rt protected from light. After freeze-drying, the crude products were dissolved in 150 µL of 300 mM NaCl and purified in a PD-10 column (Sephadex G-25) to remove any free dye. Then, the fractions

containing pure fluorescently labeled proteins were desalted by dialysis (5 × 500 mL H_2O, Spectra/Por 6, MWCO 1 kDa,) and lyophilized. The dye functionalization degrees were determined by measuring the relative UV absorbances characteristic of the proteins and fluorescent dyes, using the following extinction coefficients: GOX ε_{280} 267200 $M^{-1}cm^{-1}$, HRP ε_{405} 102000 $M^{-1}cm^{-1}$, lysozyme ε_{280} 37970 $M^{-1}cm^{-1}$, BSA ε_{280} 43824 $M^{-1}cm^{-1}$, insulin ε_{280} 5734 $M^{-1}cm^{-1}$, anti-BSA rabbit IgG ε_{280} 210000 $M^{-1}cm^{-1}$, AF488 ε_{495} 71800 $M^{-1}cm^{-1}$, Cy5 ε_{646} 250000 $M^{-1}cm^{-1}$.

GOX-AF488. From a solution of AF488-NHS (0.40 mg, 0.51 μmol, 9 eq) in dry DMSO (80 μL) and a solution of GOX (9.00 mg, 0.06 μmol) in 0.1 M $NaHCO_3$ (2.0 mL), GOX-AF488 (8.30 mg) labeled with an average of 2.3 molecules of AF488 was obtained following the General Procedure for Protein Labeling.

GOX-Cy5. From a solution of Cy5-NHS (0.96 mg, 1.44 μmol, 24 eq) in dry DMSO (80 μL) and a solution of GOX (9.00 mg, 0.06 μmol) in 0.1 M $NaHCO_3$ (2.0 mL), GOX-Cy5 (8.20 mg) labeled with an average of 2.1 molecules of Cy5 was obtained following the General Procedure for Protein Labeling.

HRP-Cy5. From a solution of Cy5-NHS (3.40 mg, 5.04 μmol, 24 eq) in dry DMSO (70 μL) and a solution of HRP (9.00 mg, 0.21 μmol) in 0.1 M $NaHCO_3$ (2.0 mL), HRP-Cy5 (8.10 mg) labeled with an average of 2.2 molecules of Cy5 was obtained following the General Procedure for Protein Labeling.

Lysozyme-Cy5. From a solution of Cy5-NHS (3.59 mg, 5.38 μmol, 11 eq) in dry DMSO (80 μL) and a solution of lysozyme (7.50 mg, 0.51 μmol) in 0.1 M $NaHCO_3$ (1.5 mL), lysozyme-Cy5 (6.80 mg) labeled with an average of 1.8 molecules of Cy5 was obtained following the General Procedure for Protein Labeling.

BSA-Cy5. From a solution of Cy5-NHS (1.12 mg, 1.68 μmol, 12 eq) in dry DMSO (100 μL) and a solution of BSA (9.00 mg, 0.14 μmol) in 0.1 M $NaHCO_3$ (1.8 mL), BSA-Cy5 (8.10 mg) labeled with an average of 0.9 molecules of Cy5 was obtained following the General Procedure for Protein Labeling.

Insulin-Cy5. From a solution of Cy5-NHS (5.50 mg, 8.24 μmol, 12 eq) in dry DMSO (80 μL) and a solution of insulin (4.10 mg, 0.71 μmol) in 0.1 M $NaHCO_3$ (1.5 mL), insulin-Cy5 (3.60 mg) labeled with an average of 0.9 molecules of Cy5 was obtained following the General Procedure for Protein Labeling.

IgG-Cy5. A solution of Cy5-NHS (0.10 mg, 0.15 μmol, 12 eq) in dry DMSO (30 μL) was added to a solution of anti-BSA rabbit IgG (1.87 mg, 0.01 μmol) in PBS pH 7.2 (2.0 mL). The reaction mixture was stirred at rt overnight protected from light and then purified by centrifugal ultrafiltration (Amicon Ultra-4 centrifugal filters 50K, 5×3.5 mL PBS, 2×3.5 mL H_2O). After freeze-drying, IgG-Cy5 (1.90 mg) was obtained with an average labeling of 1.2 molecules of Cy5.

4. Preparation and Characterization of Membranized Coacervate Microdroplets (MCM)

Solutions of Components. PEG[G3]-BA (2.16 mg/mL, 135 μM) and 3[G2]-BA (1.50 mg/mL 135 μM) were separately dissolved in 10 mM PB pH 7.0, and the resulting solutions were aged for at least 1 h at rt. Fresh solutions of Cat-CA and Cat-G were prepared in 10 mM PB pH 7.0 (Table S1). All solutions were filtered through sterile 0.22 μm nylon filters before coacervate formation. No specific measures were implemented to control catechol oxidation beyond the use of freshly prepared catechol solutions.

Table S1. Concentration of solutions of Cat-CA and Cat-G used in the preparation of coacervates with different CBA ratios.

	**CBA 1**	**CBA 2**	**CBA 3**
**Cat-CA**	0.67 mg/mL (3.65 mM)	1.33 mg/mL (7.31 mM)	2.00 mg/mL (11.00 mM)
**Cat-G**	0.85 mg/mL (3.65 mM)	1.69 mg/mL (7.31 mM)	2.54 mg/mL (11.00 mM)

General Procedure for the Preparation of MCM. For MCM prepared at different charge ratios, solutions of Cat-CA and Cat-G were mixed in different volume ratios to afford a total volume of 120 μL (Table S2). A 100 μL portion of this solution was immediately added over a solution of 3[G2]-BA (100 μL) at 21 ºC under orbital stirring (350 rpm) in an Eppendorf Thermomixer C. After 15 min of stirring, 6.2 μL of 5 M NaCl were added to afford a 150 mM NaCl concentration. The mixture was stirred for 15 min before stabilization with PEG[G3]-BA (9 μL, equivalent to 9 mol% of 3[G2]-BA). The presence of spherical MCM and the absence of aggregates were confirmed by optical microscopy. The size distribution of the samples was determined by analysis using Image-J, and their z-potential by LDA (Figure S1). Fluorescently labeled versions of MCM were prepared following the same procedure using solutions of 3[G2]-BA/3[G2]-BA-Cy5 (molar ratio 4:1) and PEG[G3]-BA/PEG[G3]-BA-FITC (molar ratio 3:1).

Table S2. Volume (μL) of Cat-CA and Cat-G solutions used in the preparation of MCM with different charge ratios.

	**Cat-CA:Cat-G charge ratio**		
	**2:1**	**1:1**	**1:2**
**Cat-CA**	80	60	40
**Cat-G**	40	60	80

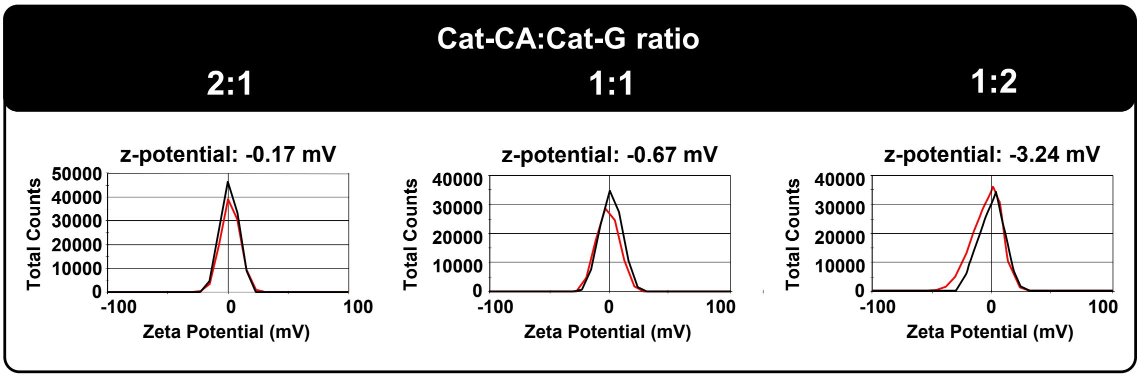

Figure S1. Z-potential measurements of MCM prepared with CBA 1 and different Cat-CA:Cat-G charge ratios.

Turbidimetry. Turbidity was determined using Eq S1, where A_{sample} and A_{buffer} are the absorbances at 600 nm of the sample and buffer, respectively, measured at 25 ºC on a Jasco V-630 or a plate reader (Tecan Infinite F200 PRO).

$$T(\%) = 100 - 10^{(2-A_{sample}+A_{buffer})} \qquad \text{Eq S1}$$

Long-Term Stability of MCM. The turbidity of MCM (1:1 Cat-CA:Cat-G ratio, CBA 1) prepared with 9 mol% of PEG[G3]-BA relative to 3[G2]-BA was studied over a period of one week. Absorbance was measured on a plate reader (Tecan Infinite F200 PRO) and turbidity calculated following Eq S1. Data was normalized to the turbidity of a freshly prepared sample (Figure S2).

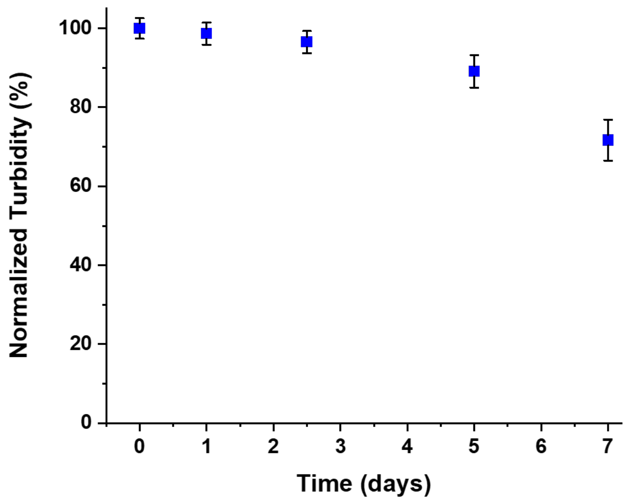

Figure S2. Normalized turbidity of MCM stabilized with PEG[G3]-BA (9 mol% relative to 3[G2]-BA) recorded for one week after preparation.

Interfacial Stabilization Studies. The turbidity of MCM (1:1 Cat-CA:Cat-G ratio, CBA 1) stabilized with different amounts of PEG[G3]-BA (1, 3, 6, 9, and 15 mol% relative to 3[G2]-BA) was analyzed 24 h after preparation and normalized to the turbidity of a freshly prepared sample with 9 mol% of PEG[G3]-BA (Figure S3). Absorbance was measured on a plate reader (Tecan Infinite F200 PRO) and turbidity calculated following Eq S1.

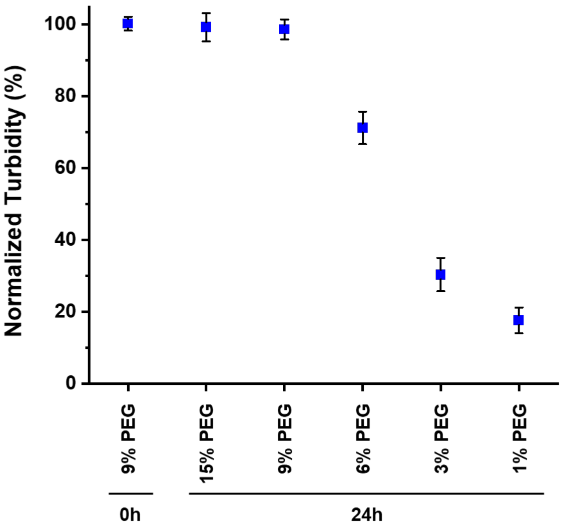

Figure S3. Turbidity of MCM stabilized with different amounts of PEG[G3]-BA (1, 3, 6, 9, and 15 mol% relative to 3[G2]-BA) recorded 24 h after preparation. Comparison with a freshly prepared MCM containing 9 mol% PEG[G3]-BA.

Ionic Strength Stability of MCM. MCM suspensions (40 μL; 1:1 Cat-CA:Cat-G ratio, CBA 1) were diluted with NaCl solutions of increasing concentration (160 μL). The resulting mixtures were shaken gently for 20 s to ensure homogeneous mixing and then were transferred to a 1 cm optical path cuvette. After 3 min, absorbance at 600 nm was measured at 25 °C on a Jasco V-630 and turbidity calculated following Eq S1 (Figure 2B).

pH-Sensitivity of MCM. MCM suspensions (50 µL; 1:1 Cat-CA:Cat-G ratio, CBA 1) were diluted using a series of buffers with different pH values (50 µL): 50 mM PB pH 7.4, and 50 mM citrate pH 6.0, 5.0, and 4.0. The turbidity of the resulting mixtures was monitored for 24 h and normalized to the turbidity of the pH 7.4 sample (Figure S4). Absorbance was measured on a plate reader (Tecan Infinite F200 PRO) and turbidity calculated following Eq S1.

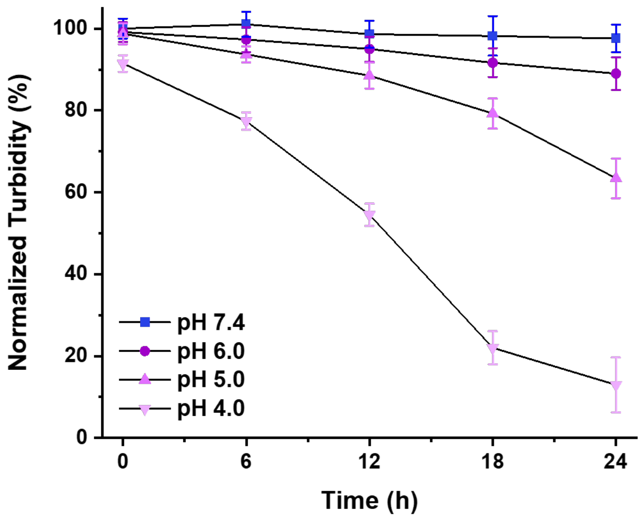

Figure S4. Normalized turbidity of MCM at different pH values analyzed for 24 h.

Dynamic Covalent Nature of the Boronate Ester Bond. NMR spectra were recorded on a Bruker NEO 750 MHz spectrometer. Chemical shifts are reported in ppm relative to the 4.79 ppm residual solvent peak for D_2O. The number of scans was 64. The acquisition time (AQ) was set at 2.75 s and the inter-scan delay (d1) at 10 s. MestReNova 14.2 software (Mestrelab Research) was used for spectral processing.

A solution of Cat-CA (250 µL, 15.96 mg/mL, 87.64 mM in D_2O, pH 7.0; CBA 6) and a solution of caffeic acid (250 µL, 15.76 mg/mL, 87.64 mM in D_2O, pH 7.0, CBA 6) were added to solutions of 3[G2]-BA (6 mg/mL, 0.54 mM in D_2O, pH 7.0, 250 µL) in separate NMR tubes (A and B in Figure 3, respectively). The disappearance of the 3[G2]-BA signals centered at 7.36 (H_{1BA}) and 3.02 ppm (H_{2BA}) was accompanied by the appearance of new peaks at 7.55 ($H_{1Cat\text{-}CA}$), 7.50 (H_{1Caff}), and 3.11 ppm ($H_{2Cat\text{-}CA}$ and H_{2Caff}). Relative integration of the H_1 and H_2 protons revealed conversions of 70% for Cat-CA and 95% for the higher affinity caffeic acid. pH values were adjusted using NaOD and DCl solutions.

To study the dynamic nature of the boronate ester, competitive ^{1}H NMR experiments were conducted (Figure 3). Solutions of caffeic acid and Cat-CA (CBA 6) were respectively added to the above A and B samples containing Cat-CA and caffeic acid esters. The immediate and quantitative formation of an identical mixed boronate ester (1:9 Cat-CA:caffeic acid ratio) was consistently observed.

Membranization Efficiency of MCM

Control Study of Micelle Formation. Equal volumes of solutions of Cat-CA (5.54 mg/mL, 30.3 mM) and Cat-G (7.02 mg/mL, 30.3 mM) in 10 mM PB pH 7.0 were mixed to afford a total volume of 350 μL. A 333 μL portion of this solution, corresponding to a CBA 3, was added to 667 μL of a solution of PEG[G3]-BA (1.50 mg/mL, 94.1 μM in 10 mM PB pH 7.0) at 21 °C under magnetic stirring. The mixture was immediately supplemented with 5 M NaCl (30 μL) up to 150 mM NaCl. After 10 min, the formation of micelles with a mean diameter of 28 nm (PDI 0.22) was confirmed by dynamic light scattering (DLS) (Figure S5).

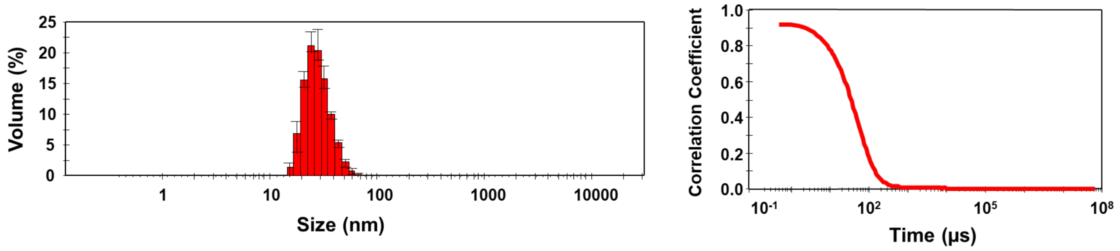

Figure S5. DLS histogram and correlation function of micelles prepared from PEG[G3]-BA and Cat-CA:Cat-G (ratio 1:1, CBA 3) recorded 10 min after formation at 25 °C.

Impact of the CBA Ratio on Membranization Efficiency. The fraction of PEG[G3]-BA assembled on the surface of the coacervate droplets was determined indirectly by measuring the fluorescence intensity of the dilute phase after centrifuging MCM samples prepared with different CBA ratios and stabilized with PEG[G3]-BA-FITC. To this end:

(a) MCM prepared with CBA ratios 1, 2, and 3 were stabilized with 9 mol% of PEG[G3]-BA:PEG[G3]-BA-FITC (2.16 mg/mL, 135 μM, molar ratio 3:1) following

the General Procedure for the Preparation of MCM described above (1:1 Cat-CA:Cat-G ratio).

(b) MCM (1:1 Cat-CA:Cat-G ratio) prepared with a CBA ratio 1 (200 μL) were stabilized with a solution of the above prepared micelles (19.44 μL) accounting for a 9 mol% of PEG[G3]-BA:PEG[G3]-BA-FITC (molar ratio 3:1). CLSM images of the resulting MCM revealed a markedly lower fluorescence intensity in the membrane compared to droplets prepared with CBA ratios 1, 2, and 3, and stabilized with 9 mol% of PEG[G3]-BA:PEG[G3]-BA-FITC (Figures 2E and S7).

The four MCM samples were left for 10 min before being centrifuged (350 g, 5 min). Then, the supernatants were diluted (1:1) with guanidinium chloride (6 M in 10 mM PB pH 7.0) and left under orbital stirring for 30 min to disassemble any micelle/aggregate. Membranization efficiencies were determined by comparing the fluorescence of the samples with a standard calibration curve made from the fluorescence emission of fresh solutions of PEG[G3]-BA-FITC of known concentrations, prepared under identical conditions (λ_{ex} 483 nm, λ_{em} 550/9 nm, plate reader Tecan Infinite F200 PRO). Membranization efficiencies of 72% PEG[G3]-BA for CBA 1, 63% for CBA 2, 43% for CBA 3, and 32% for the MCM (CBA 1) stabilized with micelles were obtained (Figure S6).

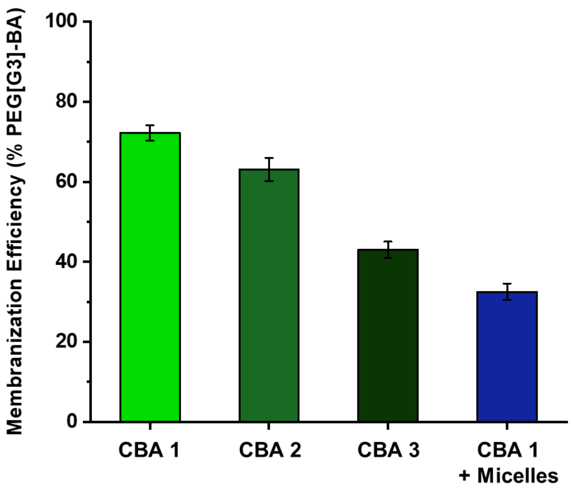

Figure S6. Membranization efficiency expressed as the percentage of PEG[G3]-BA assembled on the surface of MCM prepared with different CBA ratios (1, 2, and 3) and stabilized with 9 mol% PEG[G3]-BA:PEG[G3]-BA-FITC (molar ratio 3:1) either as free copolymer or pre-assembled into micelles.

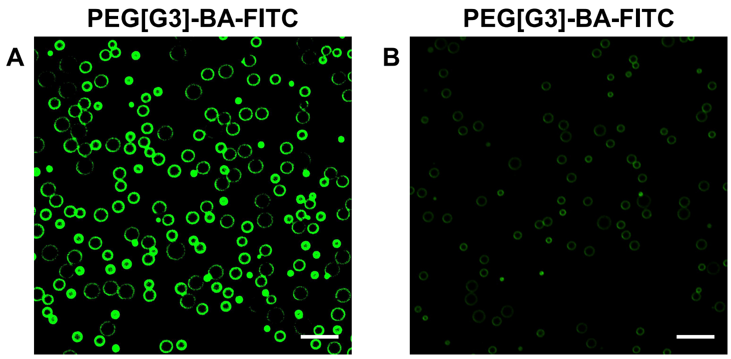

Figure S7. CLSM images of polyboronate MCM (1:1 Cat-CA:Cat-G ratio, CBA 1) interfacially stabilized with 9 mol% of PEG[G3]-BA-FITC (green) either as free copolymer (**A**) or pre-assembled into micelles (**B**). Scale bars 10 μm.

Protein-loaded MCM. Encapsulation of proteins in MCM was done by adding a solution of the corresponding fluorescently labeled protein (1.7 μL, 2 mg/mL in 10 mM PB pH 7.0) to the coacervate mixture immediately after mixing 3[G2]-BA and catechols (CBA 1). Afterwards, the General Procedure for the Preparation of MCM described above was followed. Protein encapsulation was confirmed by CLSM. Encapsulation efficiencies (EE) were obtained by comparing the fluorescence of the supernatant after centrifugation (350 *g*, 5 min) with a standard calibration curve made from the fluorescence emission of fresh solutions of the proteins of known concentration, prepared under identical conditions (λ_{ex} 622 nm, λ_{em} 692/9 nm, plate reader Tecan Infinite F200 PRO).

pEGFP-N1-loaded MCM. Encapsulation of plasmid DNA (pEGFP-N1) in MCM was done by adding a solution of the Cy5 fluorescently labeled plasmid (5.6 μL, 300 ng/μL in 10 mM HEPES pH 7.1) to the coacervate mixture immediately after mixing 3[G2]-BA and catechols (1:2 Cat-CA:Cat-G ratio, CBA 1). Afterwards, the General Procedure for the Preparation of MCM described above was followed. Plasmid encapsulation was confirmed by CLSM. A quantitative encapsulation efficiency (EE) was determined by comparing the fluorescence of the supernatant after centrifugation (350 *g*, 5 min) with a standard calibration curve made from the fluorescence emission of fresh solutions of the plasmid of known concentration, prepared under identical conditions (λ_{ex} 622 nm, λ_{em} 692/9 nm, plate reader Tecan Infinite F200 PRO).

Doped MCM. MCM doped with EPI, EGCG, and CAT were prepared by adding 5 μL of the catechol dopant solution in 10 mM PB pH 7.0 (Table S3) to 120 μL of the Cat-CA/Cat-G mixture (Table S2). The amount of catechol groups in the dopants accounts for a 10 mol% of those in the Cat-CA/Cat-G mixture. MCM were prepared following the General Procedure for the Preparation of MCM using a 1:2 Cat-CA:Cat-G ratio and CBA 1.

Table S3. Concentration of solutions of EPI, EGCG, and CAT used in the preparation of doped MCM (CBA 1).

**EPI**	2.12 mg/mL (7.31 mM)
**EGCG**	1.67 mg/mL (3.65 mM)
**CAT**	0.80 mg/mL (7.31 mM)

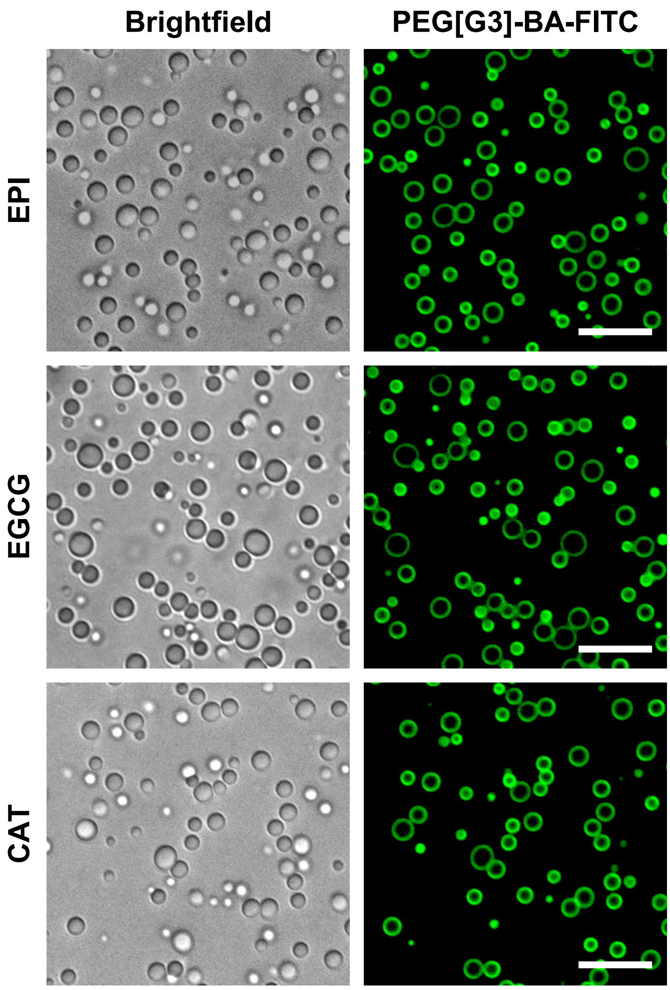

Figure S8. Brightfield and CLSM images of polyboronate MCM (1:2 Cat-CA:Cat-G ratio, CBA 1) doped with EPI, EGCG, and CAT (10 mol% catechol groups) and interfacially stabilized with PEG[G3]-BA-FITC (green). Scale bars 10 µm.

5. Enzymatic Cascade Assays

Monitoring of the Enzymatic Cascade Reaction by CLSM (Amplex Red). MCM populations (prepared at a 1:2 Cat-CA:Cat-G ratio, CBA 1; in 10 mM PB pH 7.0, 150 mM NaCl) loaded with GOX-AF488 (green) and HRP-Cy5 (blue) were mixed in a 1:1 ratio to a volume of 100 μL in a microscope slide (Cellvis 4-Chamber microwells 35mm Glass Bottom Dish with 20 mm, #1.5 cover glass). Final protein concentrations in the mixture accounted for 50 nM of GOX-AF488 and 130 nM of HRP-Cy5. A control experiment confirmed the absence of protein exchange between MCM populations for at least 3 h. Once an area of interest containing similar number of MCM from both populations was selected, an image was acquired at t = 0 after the addition of Amplex Red (3 μL, 6 μM in DMSO) to record the starting amount of resorufin present at background levels in Amplex Red. The enzymatic cascade reaction was triggered by gently pipetting glucose (3 μL, 120 μM in 10 mM PB pH 7.0) into the MCM suspension. The sample was imaged at different time points to monitor the production and localization of resorufin (red). Control experiments performed in the absence of any of the enzyme-loaded MCM (being replaced by 10 mM PB 7.0, 150 mM NaCl) did not result in the production of resorufin (Figure S9).

The individual channels were recorded sequentially using the following parameters:

- Red channel (resorufin): excitation at 561 nm; emission at 620/50 nm.
- Green channel (GOX): excitation at 488 nm; emission at 525/50 nm.
- Blue channel (HRP): excitation at 637 nm; emission at 725/40 nm.

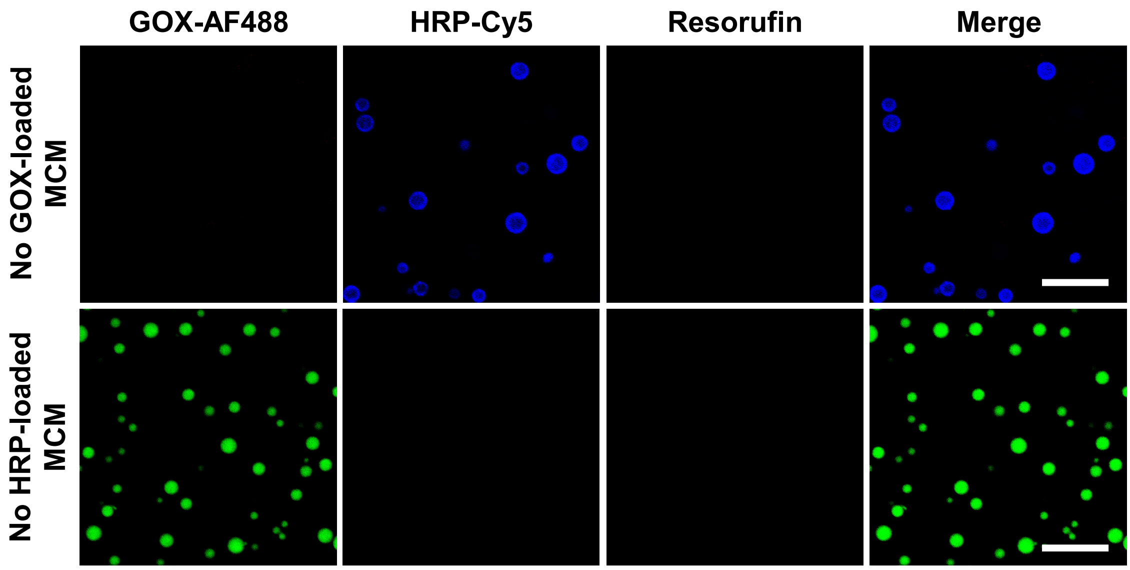

Figure S9. CLSM images (10 min) of control enzymatic cascade experiments performed without GOX- or HRP-loaded MCM. Scale bars 10 μm.

Monitoring of the Enzymatic Cascade Reaction by Fluorescence Spectroscopy (oPD). MCM populations (prepared at a 1:2 Cat-CA:Cat-G ratio, CBA 1; in 10 mM PB pH 7.0, 150 mM NaCl) loaded with GOX and HRP were centrifuged (350 *g*, 5 min) and resuspended (orbital stirring, 350 rpm) overnight in 10 mM PB pH 7.0, 150 mM NaCl to remove unencapsulated enzymes. Then, they were mixed in a 2:1 ratio to a volume of 90 μL in a 96-well microplate (Nunc F96 MicroWell flat black from Thermo Scientific) to achieve final protein concentrations of 65 nM GOX and 85 nM HRP. Then, *o*-phenylenediamine (oPD, 2 μL, 27.7 mM in 10 mM PB pH 7.0) and glucose (6 μL, 16.7 mM in 10 mM PB pH 7.0) were added stepwise to initiate the enzymatic cascade. The reaction progress was monitored in triplicate by measuring the fluorescence of the reaction product 2,3-diaminophenazine (2,3-DAP; λ_{ex} 405 nm, λ_{em} 550 nm) in a plate reader (Tecan Infinite F200 PRO). Control experiments showed no fluorescence in the absence of any of the enzyme-loaded MCM or glucose. Fluorescence data were normalized to the maximum fluorescence signal recorded during the experiment (Figure S10).

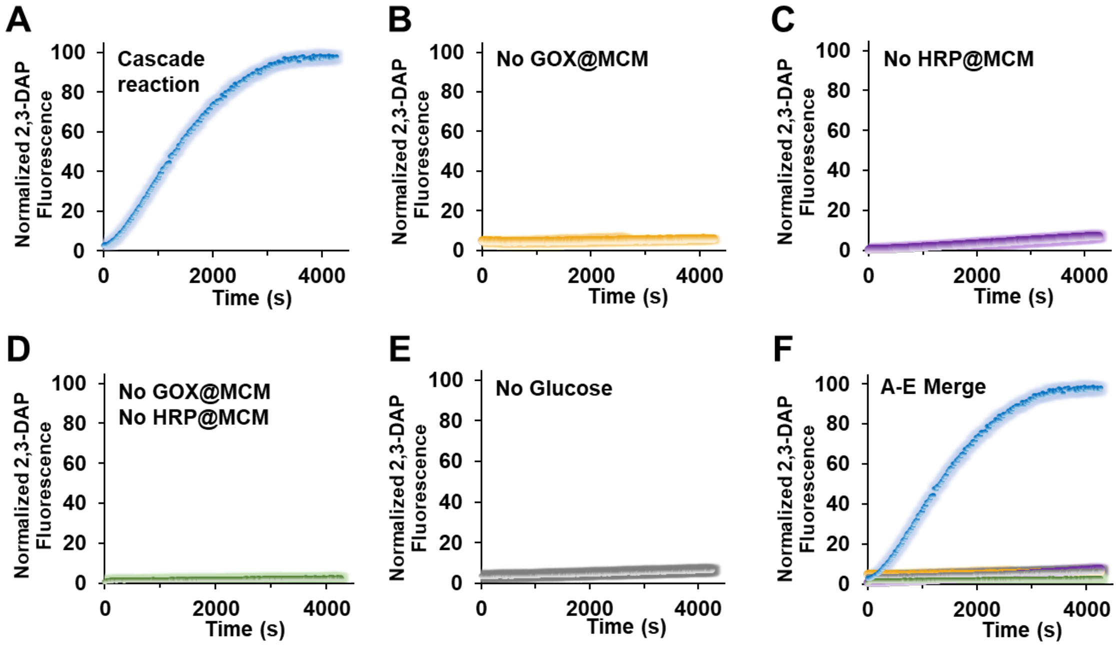

Figure S10. Progress of the enzymatic cascade between GOX@MCM and HRP@MCM (oPD as HRP substrate) studied by monitoring the fluorescence of the reaction product 2,3-DAP (**A**). Control experiments performed in the absence of GOX@MCM (**B**), HRP@MCM (**C**), GOX@MCM and HRP@MCM (**D**), or glucose (**E**). Merge of A-E (**F**). Shaded plots represent standard deviation.

6. Permeability of the MCM Membrane

Uptake/Exclusion Experiments. Sequestration of fluorescent guests – organic dyes and proteins of different charge and size – into MCM was monitored by CLSM imaging. MCM suspensions (215.2 µL in 10 mM PB pH 7.0, 150 mM NaCl; 1:1 Cat-CA:Cat-G ratio, CBA 1) were prepared in 0.5 mL Eppendorf tubes, following the above General Procedure. After 10 min, samples were transferred to 24-well plates (Cellvis, 15 mm glass bottom) and imaged by CLSM. The guests dissolved in 10 mM PB pH 7.0, 150 mM NaCl were then added and, after equilibration for 10 min, the samples were imaged again (Figures 6 and S11): methylene blue (2 µL, 1 mM; cationic, 320 g/mol), pyranine (2 µL, 1 mM; anionic, 524 g/mol), propidium iodide (2 µL, 1 mM; cationic, 668 g/mol), lysozyme-Cy5:lysozyme (4 µL, 0.5 mM, 1:0.8 molar ratio; cationic, 14 kDa), HRP-Cy5:HRP (4 µL, 0.5 mM, 1:1.2 molar ratio; cationic, 44 kDa), and GOX-Cy5:GOX (4 µL, 0.5 mM, 1:1.1 molar ratio; anionic, 160 kDa). The final concentration of guests in the MCM suspensions was 9.2 µM in all cases. Mixtures of native and fluorescently labeled proteins were used to ensure that all protein experiments were conducted with an equal degree of fluorescent labeling.

The individual channels were recorded sequentially using the following parameters:

- Pyranine: excitation at 405 nm; emission at 525/50 nm.
- Propidium iodide: excitation at 561 nm; emission at 620/50 nm.
- Cy5 & methylene blue: excitation at 637 nm; emission at 725/40 nm.

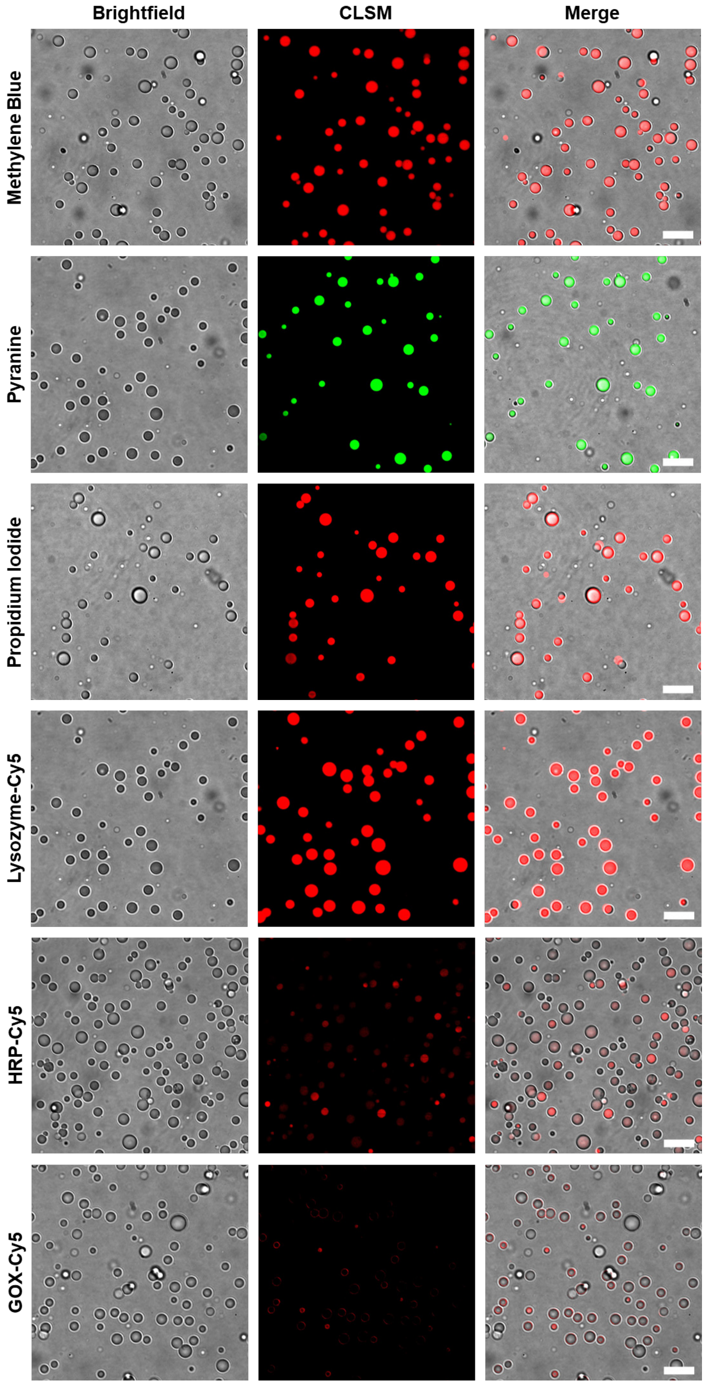

Figure S11. Permeability of the MCM membrane (Cat-CA:Cat-G 1:1, CBA 1) to different guests assessed by brightfield and CLSM imaging (10 min equilibration). Scale bars 10 μm.

Partition Coefficients. MCM suspensions (215.2 µL in 10 mM PB pH 7.0, 150 mM NaCl; 1:1 Cat-CA:Cat-G ratio, CBA 1) were prepared following the General Procedure in 0.5 mL Eppendorf tubes, the empty weights of which were determined in advance. After 10 min, fluorescent guests – organic dyes and proteins of different charge and size – were added in 10 mM PB pH 7.0, 150 mM NaCl: methylene blue (2 µL, 1 mM; cationic, 320 g/mol), pyranine (2 µL, 1 mM; anionic, 524 g/mol), propidium iodide (2 µL, 1 mM; cationic, 668 g/mol), lysozyme-Cy5 (4 µL, 0.5 mM; cationic, 14 kDa), HRP-Cy5 (4 µL, 0.5 mM; cationic, 44 kDa), and GOX-Cy5 (4 µL, 0.5 mM; anionic, 160 kDa). The final concentration of guests in the MCM suspensions was 9.2 µM in all cases. The samples were weighed and their total volume determined using the density of H_2O (1 g/mL). After 10 min of equilibration, samples were centrifuged (350 g, 5 min). The dilute phase was gently removed by pipetting – care was taken not to disturb the coacervate phase at the bottom – and transferred to a separate, pre-weighed Eppendorf tube. The volume of this dilute phase was determined using the density of H_2O (1 g/mL). The volume of the coacervate phase was determined as the difference between the initial total volume and the calculated volume of the dilute phase.

The concentration of guests in the dilute phase was determined by comparing the fluorescence intensity with a standard calibration curve made from the fluorescence emission of fresh solutions of the guests of known concentrations, prepared under identical conditions (plate reader Tecan Infinite F200 PRO). The concentration of guests in the coacervate phases was obtained by difference, considering the calculated coacervate volume. All experiments were performed in triplicate.

The uptake of guests in the MCM is described by the partition coefficient (K_p) as the ratio of concentrations in the two phases (Eq S2):

$$K_p = \frac{c_{coacervate\ phase}}{c_{dilute\ phase}} \qquad \text{Eq S2}$$

The following excitation and emission wavelengths were used for the guests: pyranine (λ_{ex} 450 nm, λ_{em} 515/9 nm), propidium iodide (λ_{ex} 540 nm, λ_{em} 622/9 nm), methylene blue (λ_{ex} 609 nm, λ_{em} 690/9 nm), and Cy5 (λ_{ex} 622 nm, λ_{em} 692/9 nm).

7. Optimizing Cytomimetic Functions of MCM by Dynamic Covalent Libraries

Fluorescence Recovery After Photobleaching (FRAP). Undoped and doped MCM prepared with 3[G2]-BA-Cy5 (1:2 Cat-CA:Cat-G ratio, CBA 1) were transferred to a μ-side 18 well glass bottom (Ibidi). FRAP experiments were performed with the FRAP interface available in the Leica LAS X software. For imaging acquisition, samples were excited with a white light laser (WLL2; λ_{ex} 649 nm, λ_{em} 654-700 nm) and visualized with an HC PL APO CS2 93×/1.30 Gly objective. An initial image was acquired to define a region of interest (ROI) with a diameter of 0.6 μm. Then, 3 images of 256 × 125 μm were acquired before the ROI was bleached using 30 iterations at 649 nm (100% laser power). Fluorescence recovery was monitored for 5 min after photobleaching by acquiring a 300 image series (0.26% laser power). Fluorescence intensities of the ROI (*S*), a reference area (a nearby coacervate not bleached, *R*), and the background (*B*) were extracted from the images using ImageJ software. Then, recovery data were normalized to background and reference area using Eq S3,[3,4,5] where *F*(*t*) is the normalized fluorescence intensity of the ROI at a given time (*t*).

$$F(t) = \frac{[S(t) - B(t)]\,[R(0) - B(0)]}{[R(t) - B(t)]\,[S(0) - B(0)]} \qquad \text{Eq S3}$$

Afterwards, *F*(t) data was fitted to a first-order exponential equation (Eq S4) using Origin 2022 software (OriginLab)

$$F(t) = A\left(1 - \mathrm{e}^{-\frac{t}{\tau}}\right) \qquad \text{Eq S4}$$

where τ is the fluorescence recovery time constant, and *A* is the amplitude of the recovery (Figure S12).

The fluorescence recovery half-life ($t_{1/2}$) was determined from Eq S5 and used to calculate the apparent diffusion coefficient (D_{app}) using Eq S6,[5,6] where ω is the radius of the ROI.

$$t_{1/2} = \tau \ln 2 \qquad \text{Eq S5}$$

$$D_{app} = 0.88\, \omega^2 / 4\, t_{1/2} \qquad \text{Eq S6}$$

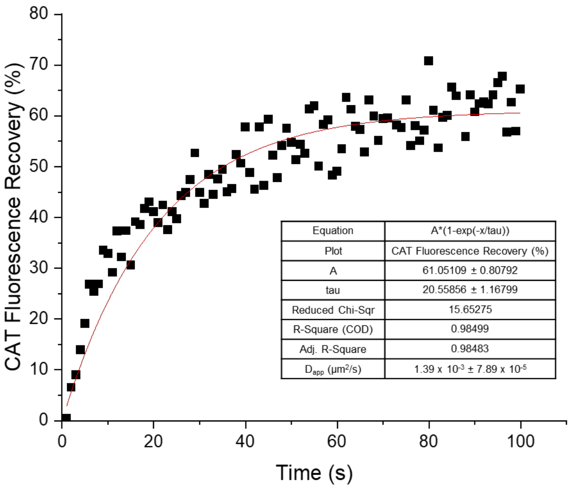

Figure S12. Fitting of the FRAP recovery curve of 3[G2]-BA-Cy5 in the CAT-doped MCM to Eq S4.

Enzymatic Cascade Activity. Independent MCM populations doped with EPI, EGCG, and CAT and encapsulating GOX and HRP were prepared following the above procedures (1:2 Cat-CA:Cat-G ratio, CBA 1). MCM were centrifuged (350 *g*, 5 min) and resuspended (orbital stirring, 350 rpm) overnight in 10 mM PB pH 7.0, 150 mM NaCl to remove unencapsulated enzymes. The EE of the enzymes were determined as described above and found to be independent of the dopant (Figure 7D). Resuspended undoped and doped MCM encapsulating GOX (60 µL) and HRP (30 µL) were placed in a 96-well microplate (Nunc F96 MicroWell flat black from Thermo Scientific) to achieve final protein concentrations of 65 nM GOX and 85 nM HRP. Then, oPD (2 µL, 27.7 mM in 10 mM PB pH 7.0) was added, followed 5 min later by glucose (6 µL, 16.7 mM in 10 mM PB pH 7.0) to initiate the enzymatic cascade. The reaction progress was monitored in triplicate by measuring the fluorescence of the reaction product 2,3-DAP (λ_{ex} 405 nm, λ_{em} 550 nm) using a plate reader (Tecan Infinite F200 PRO) (Figure 7E). Fluorescence intensities were corrected against baseline values measured from MCM suspensions before glucose addition. Despite variations in endpoint fluorescence intensity among MCM, the reaction extent was found to be independent of doping. Once the 2,3-DAP fluorescence reached a constant value at 4000 s, MCM were disassembled by addition of 1.6 M urea in MeOH (100 µL) – confirmed by brightfield microscopy as shown in Figure S13B. The fluorescence intensity of the dissolved MCM leveled off across all samples (Figure S13C), indicating a dopant-dependent quenching of 2,3-DAP fluorescence within the coacervate compartments – more pronounced for MCM with slower FRAP dynamics.

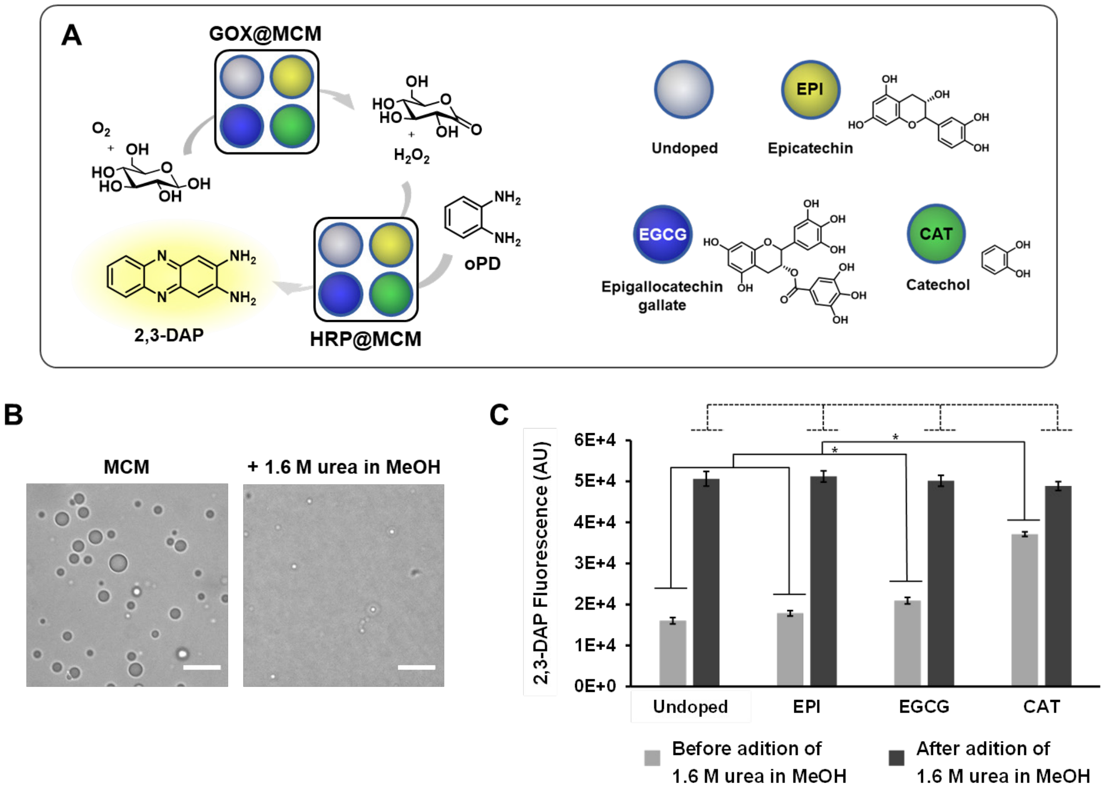

Figure S13. Enzymatic cascade and chemical communication between GOX@MCM and HRP@MCM (undoped and doped) using oPD as HRP substrate (**A**). Brightfield microscopy images show complete MCM disassembling after addition of 1.6 M urea in MeOH. Scale bars 10 µm. (**B**). 2,3-DAP fluorescence intensity in undoped and doped MCM (cascade reactions at 4000 s) level off after addition of 1.6 M urea in MeOH (intensities of dark grey columns were corrected for dilution). (*) indicates statistical difference ($p < 0.05$) analyzed by one-way ANOVA, followed by a Tukey multiple comparisons test (**C**).

Initial Reaction Rates. Initial reaction rates (v_0) were determined from cascade reaction experiments with undoped and doped MCM, performed as described in the previous section "Enzymatic Cascade Activity". Experiments were done in triplicate, and v_0 was calculated by linear fitting of the time-dependent 2,3-DAP fluorescence over the first 20 s of reaction (the slope of the lines represent v_0 in $RFU \cdot s^{-1}$). The maximum fluorescence intensities in these experiments accounted for less than 5% of the endpoint values reached after 4000 s (Figure 7E).

To express v_0 in $M \cdot s^{-1}$ units, 2,3-DAP fluorescence intensities (RFU) were converted to concentration values. To this end, the fluorescence intensities of undoped and doped MCM mixtures of GOX@MCM (60 μL) and HRP@MCM (30 μL), supplemented with known concentrations of 2,3-DAP (10 μL in 10 mM PB pH 7.0, 150 mM NaCl), were compared with a calibration curve made with freshly prepared solutions of known concentrations of 2,3-DAP in 10 mM PB pH 7.0, 150 mM NaCl (fluorescence intensities were corrected against baseline values measured from MCM suspensions and buffer before 2,3-DAP addition, respectively). As seen in Figure S14, the slopes of the 2,3-DAP fluorescence-concentration plots were nearly identical for undoped and doped MCM and matched the calibration curve of free 2,3-DAP in buffer, indicating no fluorescence quenching within MCM in the early stages of the cascade reaction. Using this information, linearized plots of 2,3-DAP concentration versus time were generated for the enzymatic cascade in undoped and doped MCM (Figure S15). The slopes of these lines represent v_0 in $\mu M \cdot s^{-1}$.

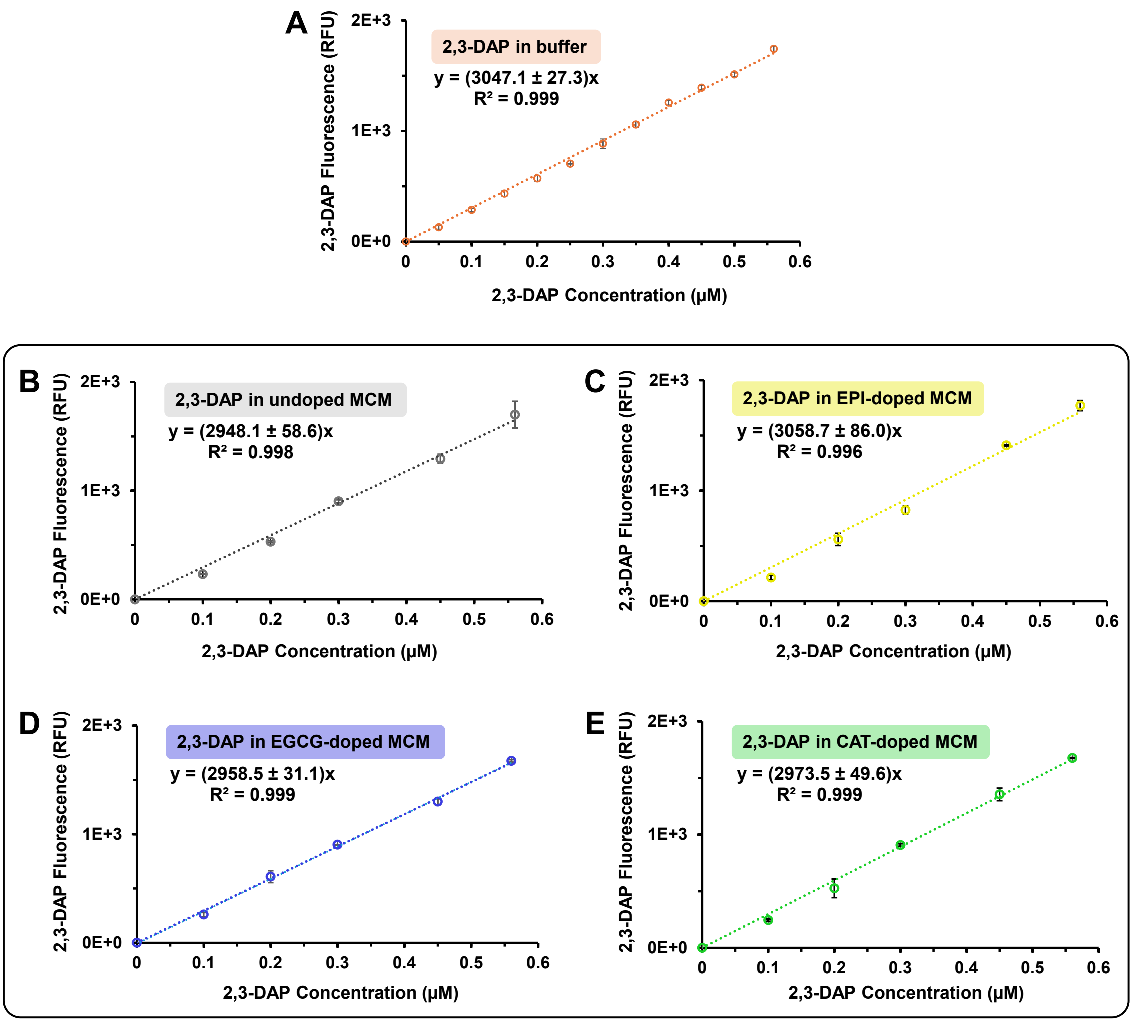

Figure S14. Linearized plots of 2,3-DAP fluorescence versus concentration in 10 mM PB pH 7.0, 150 mM NaCl (**A**), undoped MCM (**B**), EPI-doped MCM (**C**), EGCG-doped MCM (**D**), and CAT-doped MCM (**E**).

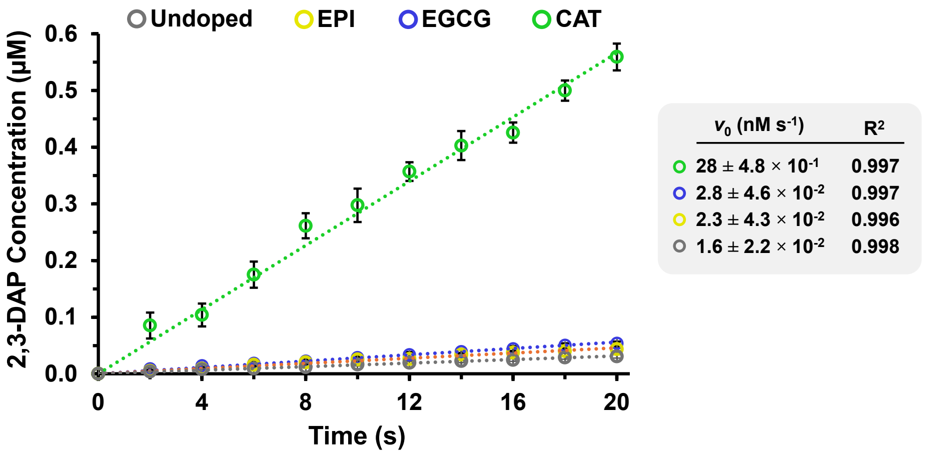

Figure S15. Linearized plots of 2,3-DAP concentration versus time for the GOX-HRP enzymatic cascade performed in undoped and doped MCM ($R^2 \geq 0.996$ in all cases). The slopes of the lines represent v_0 (see inserted Table). A 17.5-fold increase in reaction kinetics is shown when doping MCM with CAT.

8. Cell Viability Studies

Stability of MCM in Complete Cell Culture Medium. MCM (1:1 Cat-CA:Cat-G ratio, CBA 1) stabilized with 9 mol% of PEG[G3]-BA:PEG[G3]-BA-FITC (molar ratio 3:1) were mixed with an equal volume of Dulbecco's modified Eagle's medium (DMEM) with high glucose, containing 10% fetal bovine serum (FBS) and supplemented with 50 U/mL penicillin and 50 U/mL streptomycin. The sample was left under orbital stirring for 6 h before microscopy analysis.

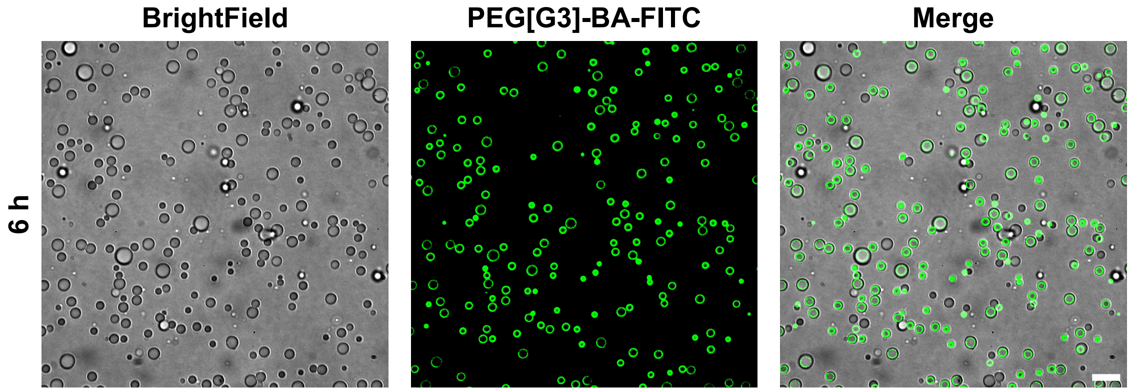

Figure S16. Brightfield and CLSM images of polyboronate MCM (1:1 Cat-CA:Cat-G ratio, CBA 1) interfacially stabilized with PEG[G3]-BA-FITC (green) after 6 h in complete cell culture medium. Scale bar 10 µm.

A549 Culture. Human adenocarcinoma alveolar basal epithelial (A549) cells, obtained from the European Collection of Authenticated Cell Cultures (ECACC), were cultured at 37 °C in a 5% CO_2 atmosphere in DMEM with high glucose, containing 10% FBS and supplemented with 50 U/mL penicillin and 50 U/mL streptomycin, in 75 cm^2 cell culture flasks. This modified DMEM is referred in the text as "medium".

Cell Viability. Undoped MCM prepared following the General Procedure described above (1:1 Cat-CA:Cat-G ratio, CBA 1) at a concentration of 1.17 mg/mL in 10 mM PB pH 7.0, 150 mM NaCl were diluted with medium to reach final concentrations between

300 and 15 μg/mL. A549 cells were seeded in 96-well plates at a density of 100000 cells/mL and incubated at 37 °C in 5% CO_2 for 24 h. Then, the medium was replaced with the MCM solutions (100 μL) and incubation continued for 24 or 72 h. Cell viability was determined by a colorimetric assay with CCK-8 following the manufacturer's protocol. After 2 h of incubation with a 6% solution of CCK-8 in fresh medium, supernatant solution was transferred to a 96-well culture plate (Corning Costar 96-Well microplates from Thermo Scientific). Viability was determined by measuring the supernatant absorbance at 450 nm in a plate reader Tecan Infinite F200 PRO. Absorbance (A) from a 6% CCK-8 solution in medium was subtracted from all data points. Viability was calculated as follows:

$$\text{Cell Viability (\%)} = 100 \times \frac{(A_{\text{sample}} - A_{6\%\ \text{CCK-8}})}{(A_{\text{control}} - A_{6\%\ \text{CCK-8}})} \qquad \text{Eq S7}$$

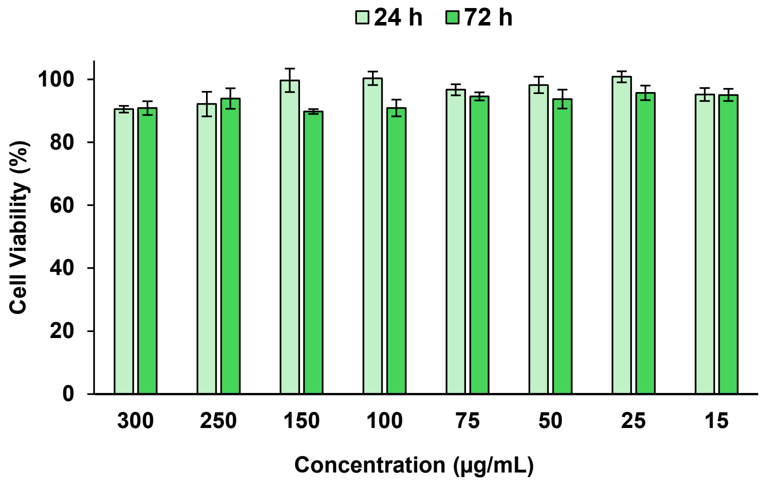

Figure S17. Cell viability (CCK-8, 24 and 72 h) of A549 cells in the presence of undoped MCM.

9. References

1. Delgado Gonzalez, B.; Lopez-Blanco, R.; Parcero-Bouzas, S.; Barreiro-Piñeiro, N.; Garcia-Abuin, L.; Fernandez-Megia, E. Dynamic Covalent Boronate Chemistry Accelerates the Screening of Polymeric Gene Delivery Vectors via In Situ Complexation of Nucleic Acids. *J. Am. Chem. Soc.* **2024**, *146*, 17211-17219.

2. Zhao, A. Y.; Brooks, A. F.; Raffel, D. M.; Stauff, J.; Arteaga, J.; Scott, P. J. H.; Shao, X. Fully Automated Radiosynthesis of [^{11}C]Guanidines for Cardiac PET Imaging. *ACS Med. Chem. Lett.* **2020**, *11*, 2325-2330.

3. Phair, R. D.; Gorski, S. A.; Misteli, T. In *Methods Enzymol.*; Academic Press: 2003; Vol. 375, p 393-414.

4. Jia, T. Z.; Hentrich, C.; Szostak, J. W. Rapid RNA Exchange in Aqueous Two-Phase System and Coacervate Droplets. *Orig. Life Evol. Biosph.* **2014**, *44*, 1-12.

5. Aumiller, W. M.; Pir Cakmak, F.; Davis, B. W.; Keating, C. D. RNA-Based Coacervates as a Model for Membraneless Organelles: Formation, Properties, and Interfacial Liposome Assembly. *Langmuir* **2016**, *32*, 10042-10053.

6. Axelrod, D.; Koppel, D. E.; Schlessinger, J.; Elson, E.; Webb, W. W. Mobility measurement by analysis of fluorescence photobleaching recovery kinetics. *Biophys J.* **1976**, *16*, 1055-1069.
